# A dynamic mucin mRNA signature associates with COVID-19 disease presentation and severity

**DOI:** 10.1172/jci.insight.151777

**Published:** 2021-10-08

**Authors:** Annemieke Smet, Tom Breugelmans, Johan Michiels, Kevin Lamote, Wout Arras, Joris G. De Man, Leo Heyndrickx, Anne Hauner, Manon Huizing, Surbhi Malhotra-Kumar, Martin Lammens, An Hotterbeekx, Samir Kumar-Singh, Aline Verstraeten, Bart Loeys, Veronique Verhoeven, Rita Jacobs, Karolien Dams, Samuel Coenen, Kevin K. Ariën, Philippe G. Jorens, Benedicte Y. De Winter

**Affiliations:** 1 Laboratory of Experimental Medicine and Pediatrics, Faculty of Medicine and Health Sciences, and; 2 Infla-med, Centre of Excellence, University of Antwerp, Antwerp, Belgium.; 3 Virology Unit, Institute of Tropical Medicine Antwerp, Antwerp, Belgium.; 4 Internal Medicine and Pediatrics, Ghent University, Ghent, Belgium.; 5 Biobank Antwerpen, Antwerp University Hospital, Edegem, Belgium.; 6 Laboratory of Medical Microbiology, Vaccine and Infectious Disease Institute, Faculty of Medicine and Health Sciences, University of Antwerp, Antwerp, Belgium.; 7 Department of Histopathology, Antwerp University Hospital, Edegem, Belgium.; 8 Laboratory of Cell Biology and Histology, Molecular Pathology Group, Faculty of Medicine and Health Sciences, University of Antwerp, Antwerp, Belgium.; 9 Center of Medical Genetics, University of Antwerp and Antwerp University Hospital, Antwerp, Belgium.; 10 Department of Family Medicine and Population Health, Faculty of Medicine and Health Sciences, University of Antwerp, Antwerp, Belgium.; 11 Critical Care Medicine, Antwerp University Hospital, Edegem, Belgium.; 12 Department of Biomedical Sciences, University of Antwerp, Antwerp, Belgium.; 13 Division of Gastroenterology and Hepatology, Antwerp University Hospital, Edegem, Belgium.

**Keywords:** COVID-19, Innate immunity, Molecular biology

## Abstract

**BACKGROUND:**

SARS-CoV-2 infection induces mucin overexpression, further promoting disease. Given that mucins are critical components of innate immunity, unraveling their expression profiles that dictate the course of disease could greatly enhance our understanding and management of COVID-19.

**METHODS:**

Using validated RT-PCR assays, we assessed mucin mRNA expression in the blood of patients with symptomatic COVID-19 compared with symptomatic patients without COVID-19 and healthy controls and correlated the data with clinical outcome parameters. Additionally, we analyzed mucin expression in mucus and lung tissue from patients with COVID-19 and investigated the effect of drugs for COVID-19 treatment on SARS-CoV-2–induced mucin expression in pulmonary epithelial cells.

**RESULTS:**

We identified a dynamic blood mucin mRNA signature that clearly distinguished patients with symptomatic COVID-19 from patients without COVID-19 based on expression of *MUC1*, *MUC2*, *MUC4*, *MUC6*, *MUC13*, *MUC16*, and *MUC20* (AUC_ROC_ of 91.8%; sensitivity and specificity of 90.6% and 93.3%, respectively) and that discriminated between mild and critical COVID-19 based on the expression of *MUC16, MUC20,* and *MUC21* (AUC_ROC_ of 89.1%; sensitivity and specificity of 90.0% and 85.7%, respectively). Differences in the transcriptional landscape of mucins in critical cases compared with mild cases identified associations with COVID-19 symptoms, respiratory support, organ failure, secondary infections, and mortality. Furthermore, we identified different mucins in the mucus and lung tissue of critically ill COVID-19 patients and showed the ability of baricitinib, tocilizumab, favipiravir, and remdesivir to suppress expression of SARS-CoV-2–induced mucins.

**CONCLUSION:**

This multifaceted blood mucin mRNA signature showed the potential role of mucin profiling in diagnosing, estimating severity, and guiding treatment options in patients with COVID-19.

**FUNDING:**

The Antwerp University Research and the Research Foundation Flanders COVID-19 funds.

## Graphical abstract

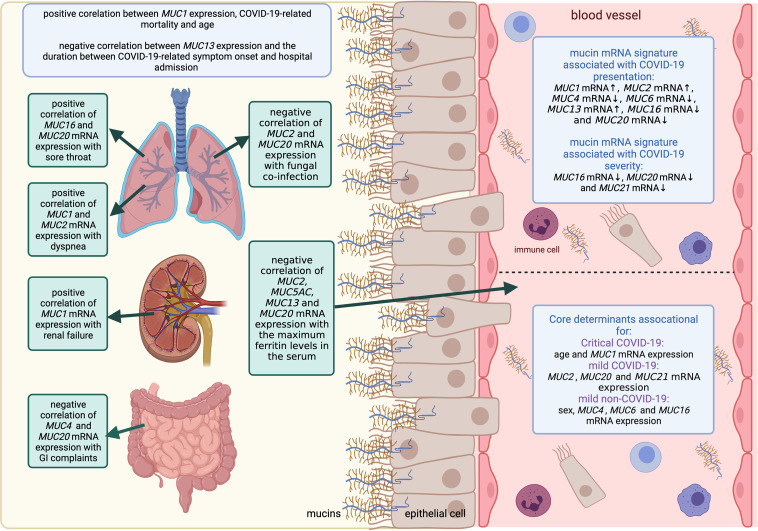

## Introduction

A novel coronavirus, SARS-CoV-2, causing COVID-19, emerged in Wuhan, China, in December 2019 and has since then disseminated globally. Patients with COVID-19 exhibit a broad spectrum of disease severity, with 80% showing no or mild to moderate symptoms, including fever, dry cough, fatigue, shortness of breath, and changes in smell or taste, whereas gastrointestinal symptoms, such as diarrhea, abdominal pain, loss of appetite, and nausea, can also occur (1–3). Fifteen percent of individuals show severe symptoms and 5% develop acute lung injury with a potential progression toward lethal acute respiratory distress syndrome (ARDS) and multiple organ failure (2). In addition to elderly individuals or those with chronic underlying diseases, young, healthy individuals and even children die of COVID-19, albeit less frequently (1, 3, 4). This underscores the urgent need to unravel molecular factors that shape the course of COVID-19 and identify patients at risk for progressing to severe disease.

Respiratory ciliated epithelial cells are the primary targets of SARS-CoV-2, and viral entry requires binding to the ACE2 receptor and subsequent priming by TMPRSS2. Interestingly, ACE2 expression increases with age and variation in ACE2 expression between children with high and low viral loads has recently been described (4, 5). However, given that other coronaviruses with markedly milder pathogenicity also use ACE2 for initial cellular entry (5), we can speculate that SARS-CoV-2 uses additional factors mediating infection of ACE2^+^ cells and subsequent tissue damage.

Secreted and transmembrane mucins produced by goblet and ciliated cells, respectively, are the gatekeepers of the mucus layer protecting the epithelial cells lining the respiratory, gastrointestinal, and reproductive tracts against injurious substances (6). Besides having a protective function, transmembrane mucins also participate in intracellular signal transduction and thus play an important role in the homeostasis and survival of epithelial cells (7). Upon disease, however, aberrant mucin expression forms a dysfunctional mucus barrier and becomes pathological (8). Indeed, mucin hypersecretion is a major clinical feature seen in severely ill patients with COVID-19; mucus accumulation in the airways obstructs the respiratory tract and complicates breathing and recovery (9–11). A few studies investigating a small number of patients have reported aberrant expression of several mucins, including MUC2, MUC4, MUC5AC, MUC5B, MUC6, MUC13, MUC16, and MUC20, in the mucus or BALF of severely ill patients with COVID-19 (9–11). Furthermore, inappropriate overexpression of transmembrane mucins can affect epithelial barrier integrity by disrupting cell polarity and cell-cell interactions, resulting in tight junction dysfunction (8). These observations prompted us to hypothesize that SARS-CoV-2 infection induces mucin overexpression and subsequent epithelial barrier dysfunction, further promoting disease. Unraveling the mucin expression profiles in patients with COVID-19 with varying degrees of disease severity will thus generate more insights into the key roles of these glycoproteins in the course of COVID-19. Given that epithelial cells can enter the bloodstream because of mucosal barrier injury, as shown for other inflammatory diseases (12–16), the peripheral blood may provide a unique tool to monitor the levels of shed mucins among different patient groups, including during COVID-19. Recent transcriptomic analyses of COVID-19 blood samples have reported the presence of mucin mRNA, further underlining the suitability of blood samples to measure mucin mRNA expression (17). Underlying microlesions may contribute to the circulation of epithelial cells in seemingly healthy individuals, allowing measurement of mucin expression in controls, albeit at low-level detection (12). In addition to their tropism for epithelial cells, some mucins can additionally be expressed by immune cells circulating in the bloodstream as well (13, 18, 19).

Furthermore, a dysfunctional mucus layer caused by SARS-CoV-2–induced mucin hypersecretion is difficult to treat with drugs, highlighting the clinical value of attacking/normalizing mucins at the earlier stage of the disease (10, 11, 20). However, whether and how experimental treatments approved for COVID-19 affect mucin expression has not been investigated.

To address the above research questions regarding mucin expression in COVID-19, we applied validated RT-PCR assays integrated with clinical data to examine the transcriptional mucin landscape in the blood of patients with symptomatic COVID-19, symptomatic patients without COVID-19, and healthy controls and investigated the effect of COVID-19 drugs on SARS-CoV-2–induced mucin expression in pulmonary epithelial cells. Here, we identified a mucin mRNA signature associated with COVID-19 presentation and severity along with the ability of baricitinib (JAK inhibitor), tocilizumab (IL-6R inhibitor), favipiravir (RNA-dependent RNA polymerase [RdRp] inhibitor), and remdesivir (nucleoside triphosphate analogue inhibiting [RdRp]) to suppress mucin hypersecretion and more specifically the mucins defining this dynamic signature.

## Results

### Patient demographics and clinical characteristics.

**Figure 1:**
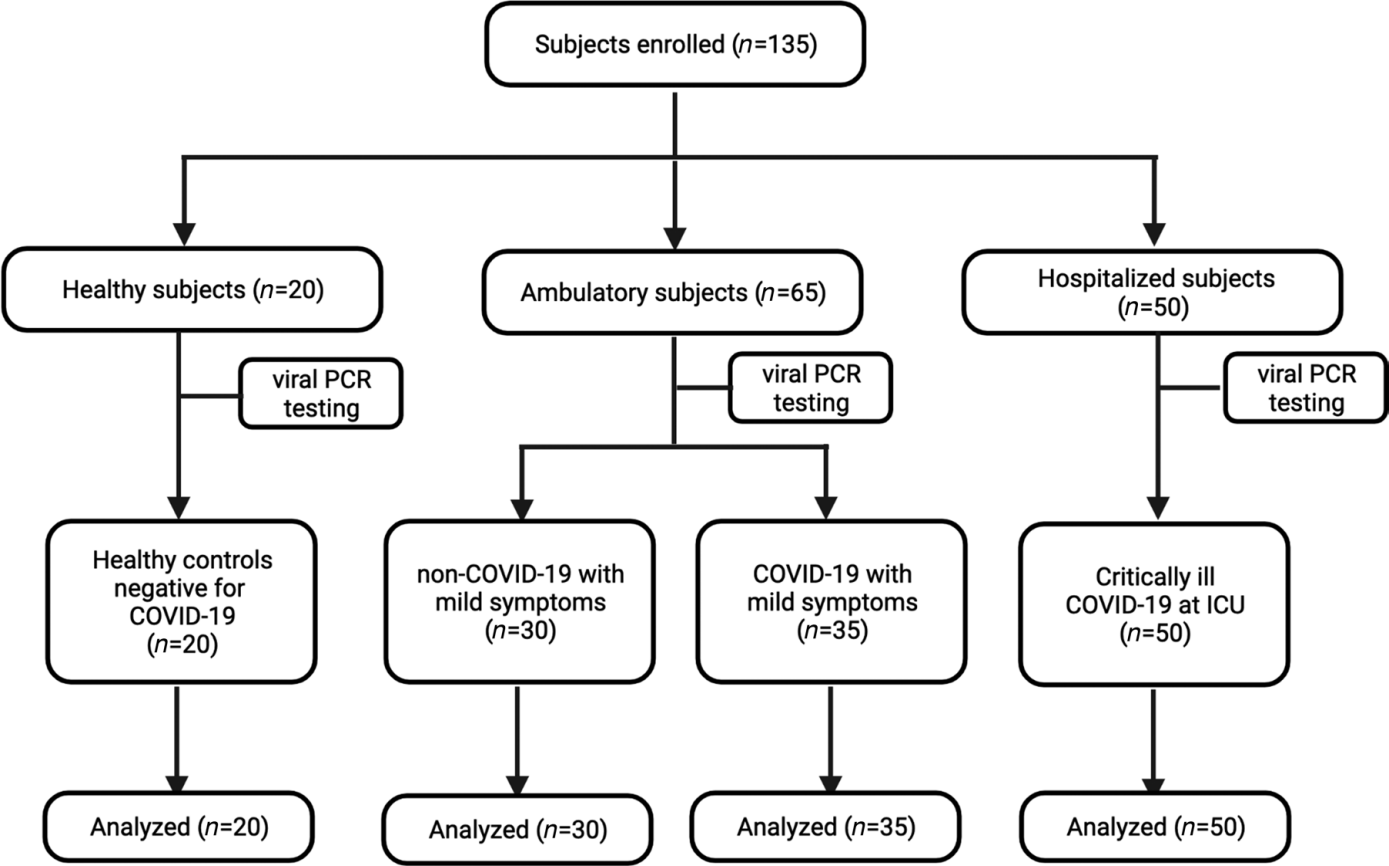
CONSORT flow diagram for participants enrolled in the study.

In total, we included 135 individuals divided into 4 patient groups (Figure 1); 85 had confirmed COVID-19 infection: the critically ill COVID-19 group consisting of 50 patients with severe symptoms (severe hypoxemia, ARDS) necessitating intensive care unit (ICU) admission and the mild COVID-19 group consisting of 35 ambulatory patients with no (*n* = 3), mild (*n* = 28), or moderate (*n* = 4) symptoms. Thirty ambulatory patients, designated as the mild non–COVID-19 patient group, had common cold–like symptoms and were screened negative for COVID-19. The 20 individuals assigned to the healthy control group were negative for COVID-19 and were confirmed as asymptomatic. The demographic and clinical characteristics of all patient groups are summarized in Table 1. More males than females were recruited in the critically ill COVID-19 group that had a median age of 65 years (Table 1), whereas the ambulatory mild COVID-19 and non–COVID-19 patient groups comprised more females than males and were significantly younger, having a median age of 34 and 36 years, respectively (Table 1). The healthy control group consisted of slightly more males than females and encompassed all ages of the 3 symptomatic patient groups (Table 1). Information on symptoms upon emergency admission (critically ill COVID-19 group) or PCR testing (ambulatory patient groups) was also available. Overall, symptoms described by critically ill COVID-19 patients included cough (32%), dyspnea (48%), fever (32%), gastrointestinal complaints (30%), and malaise (28%; Table 1). On the contrary, the majority of the ambulatory COVID-19 patients experienced loss of smell and taste (i.e., anosmia/ageusia, 45.7%), cough (37.2%), fever (34.3%), headache (37.2%), and rhinitis (37.2%; Table 1). Similar symptoms were also described in the ambulatory COVID-19–negative group except for loss of smell and taste (0%; Table 1).

Regarding hospitalization of the critically ill COVID-19 patients, the median duration from symptom onset until hospital admission was 6.5 days, with a total median hospitalization of 22 days, of which about 14 days were at the ICU (Table 1). All patients received respiratory support and the median ratio of minimal partial pressure of arterial oxygen to fractional concentration of oxygen inspired (P_a_O_2_/F_i_O_2_) was 79 mmHg. Thirty-four ICU patients (68%) required invasive ventilation with a median length of 10 days, of which half of them (*n* = 16) also needed a replacement of the endotracheal tube (ETT) due to mucus obstruction, among other factors (Table 1). Furthermore, the maximum sequential organ failure assessment (SOFA) score was 12, whereas the maximum IL-6 and ferritin serum levels were 102 pg/mL and 1628 μg/l, respectively. Other clinical outcome parameters, such as the occurrence of secondary coinfections, neurological, cardiac, and thromboembolic complications and in-hospital mortality, are also shown in Table 1. Finally, chronic medical conditions, such as hypertension (40%), lung disease (26%), diabetes (18%), obesity (48%), and malignancy (22%) were the most common comorbidities in the critically ill COVID-19 patients (Table 1), and almost all patients (96%) were treated with dexamethasone, of which a minority (8%) received cotreatment with remdesivir or tocilizumab.

### A peripheral blood mucin mRNA signature associated with COVID-19.

**Figure 2:**
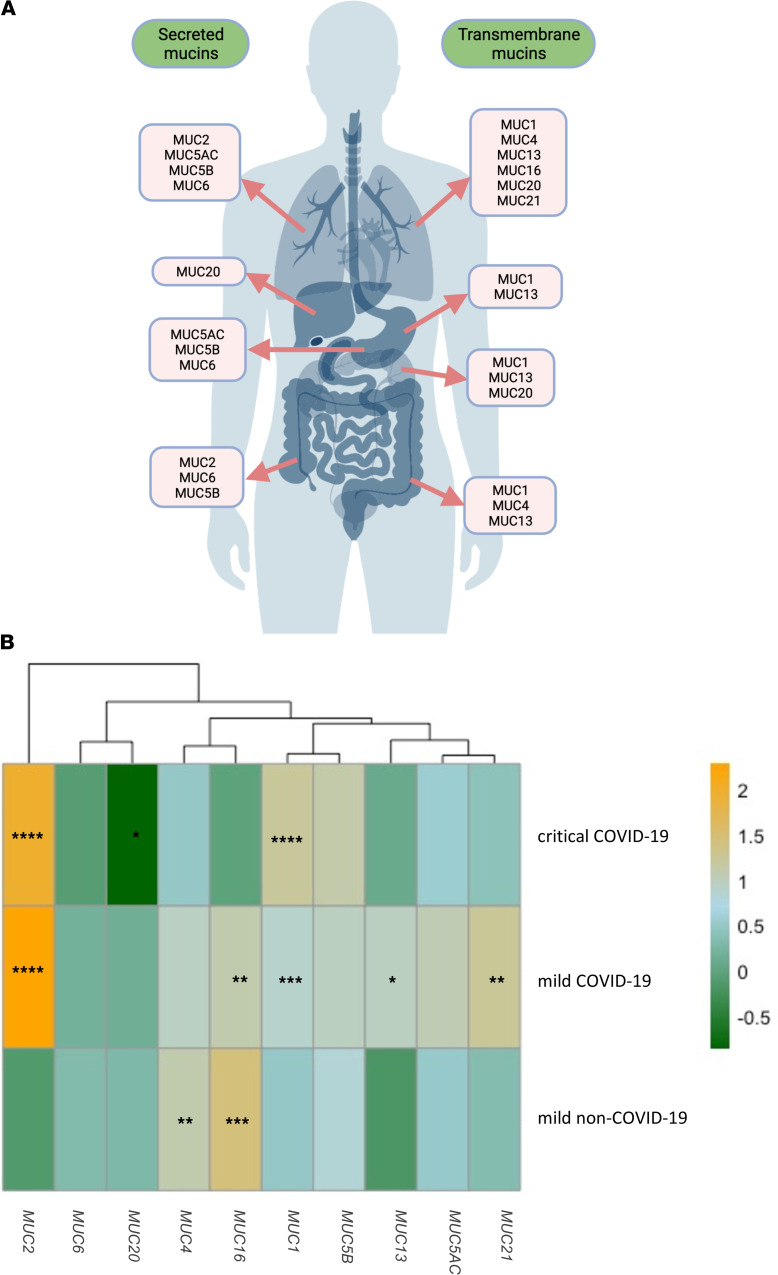
Distribution of secreted and transmembrane mucins at the respiratory (trachea and lungs), gastrointestinal (stomach, intestines, and liver), and urogenital tracts (kidneys) and the peripheral transcriptional mucin signature in critically ill COVID-19, mild COVID-19, and mild non–COVID-19 patients. (**A**) Schematic representation of the transmembrane and secreted mucins analyzed in this study and known to be expressed at the mucosal surfaces of the human body (i.e., the respiratory, gastrointestinal, and urogenital tracts) (6). (**B**) Heatmap showing the log_2_ fold-change mucin expression data in the different patient cohorts, i.e., critical COVID-19 (*n* = 50), mild COVID-19 (*n* = 35), mild non–COVID-19 (*n* = 30) normalized to the mucin expression data in healthy controls and clustered by expression. Significant differences between critically ill COVID-19, mild COVID-19, and non–COVID-19 patients and healthy controls are indicated by *P < 0.05; **P < 0.01, ***P < 0.001, ****P < 0.0001. One-way ANOVA, Tukey’s post hoc multiple-comparison test.

**Figure 3:**
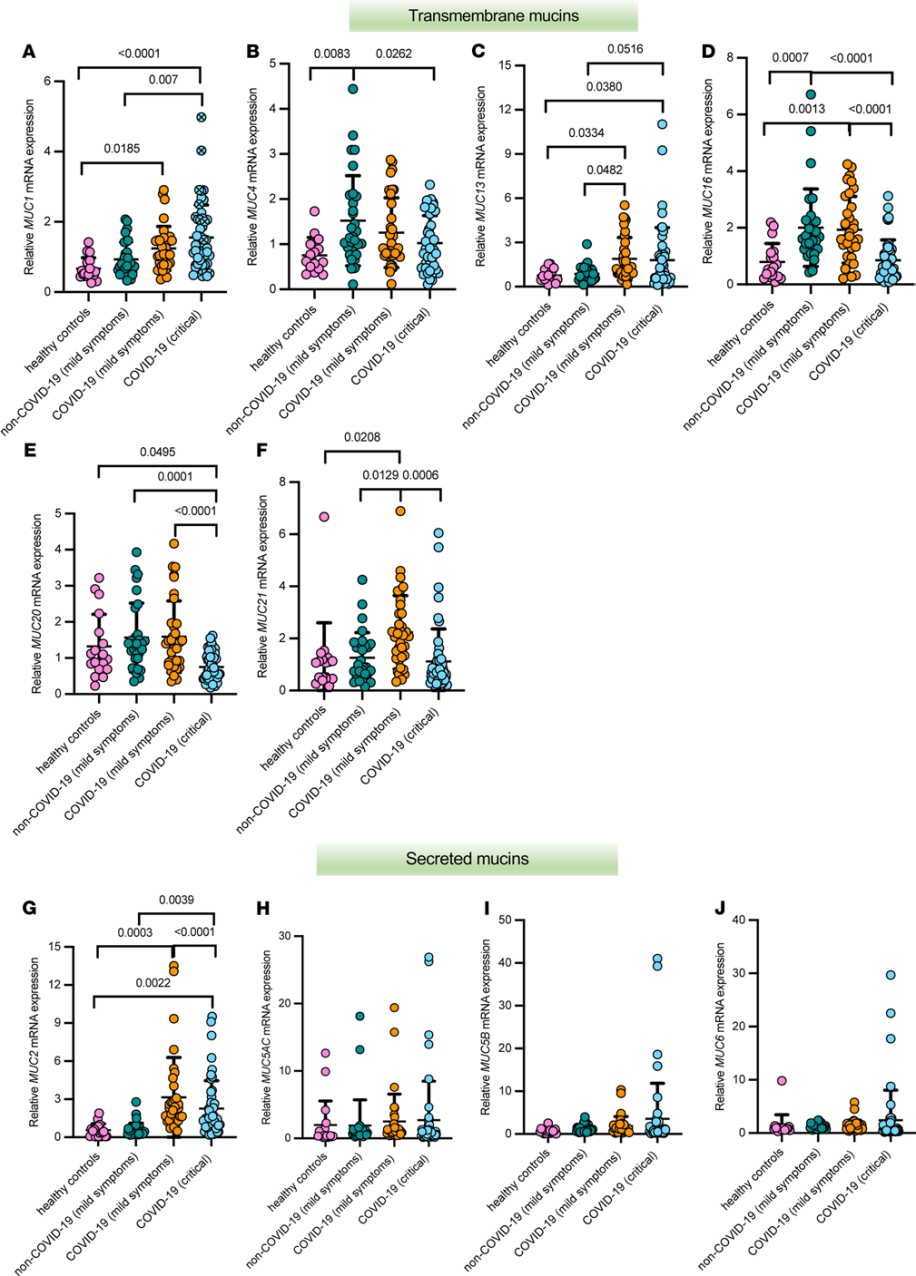
Relative transcriptional landscape of mucins in the blood of patients with critical COVID-19, mild COVID-19, and mild non–COVID-19 and healthy controls. Relative mRNA expression of *MUC1* (**A**), *MUC4* (**B**), *MUC13* (**C**), *MUC16* (**D**), *MUC20* (**E**), *MUC21* (**F**), *MUC2* (**G**), *MUC5AC* (**H**), *MUC5B* (**I**), and *MUC6* (**J**) in critically ill COVID-19 (*n* = 50), mild COVID-19 (*n* = 35), mild non–COVID-19 (*n* = 30) and healthy controls (*n* = 20). Data are presented as mean ± SEM. Each filled circle in the dot plots represents a patient and a blue filled circle with a cross (*n* = 14), as shown for MUC1, denotes deceased patients in the critically ill COVID-19 group. Significant differences between critically ill COVID-19, mild COVID-19, and non–COVID-19 patients and healthy controls as well as among the patient groups are indicated by a *P* value. One-way ANOVA, Tukey’s post hoc multiple-comparison test.

First, we tested the blood samples from the different patient groups to measure mRNA expression of transmembrane and secreted mucins known to be expressed in the respiratory, gastrointestinal, and urogenital tracts, as shown in Figure 2A (6). Mucin mRNA expression data is either presented as log_2_-transformed fold-changes compared with healthy controls (Figure 2B and Supplemental Figure 1; supplemental material available online with this article; https://doi.org/10.1172/jci.insight.151777DS1) or as relative mRNA expression values (Figure 3, A–J) for each group individually. *MUC1* mRNA expression was significantly higher in the blood of critically ill patients with COVID and those with mild COVID-19 compared with healthy controls. A significant difference in *MUC1* mRNA expression was also seen between the critically ill COVID-19 patients and the mild non–COVID-19 patient group (Figure 2B, Figure 3A, and Supplemental Figure 1A). Compared with healthy controls and the mild non–COVID-19 group, expression of *MUC2* mRNA was significantly altered in the critically ill patients with COVID and those with mild COVID-19 (Figure 2B, Figure 3G, and Supplemental Figure 1B). Interestingly, expression of *MUC4* mRNA was significantly lower in the critically ill COVID-19 group compared with the mild patient groups and was significantly increased in the mild non–COVID-19 group compared with healthy controls (Figure 2B, Figure 3B, and Supplemental Figure 1A). A significant increase in *MUC13* mRNA expression was seen in the COVID-19 patient groups compared with healthy controls and the mild non–COVID-19 group, whereas *MUC21* mRNA expression was only significantly increased in the mild COVID-19 patient group (Figure 2B, Figure 3, C and F, and Supplemental Figure 1A). On the contrary, mRNA expression of *MUC16* was significantly increased in the mild patient groups compared with controls and the critically ill COVID-19 patients, whereas expression of *MUC20* mRNA was significantly decreased in the critically ill COVID-19 group compared with controls and the mild patient groups (Figure 2B, Figure 3, D and E, and Supplemental Figure 1A). No significant alterations in mRNA expression were identified for *MUC5AC*, *MUC5B*, and *MUC6* (Figure 2B, Figure 3 H–J, and Supplemental Figure 1B). Besides, most of these mucins could also be detected in the mucus accumulating in the airways of critically ill COVID-19 patients (Supplemental Figure 2), suggesting that the mucin mRNA expression levels measured in the bloodstream could possibly be due to mucin hypersecretion and subsequent breaches in the respiratory mucosal barrier triggered by SARS-CoV-2.

**Figure 4:**
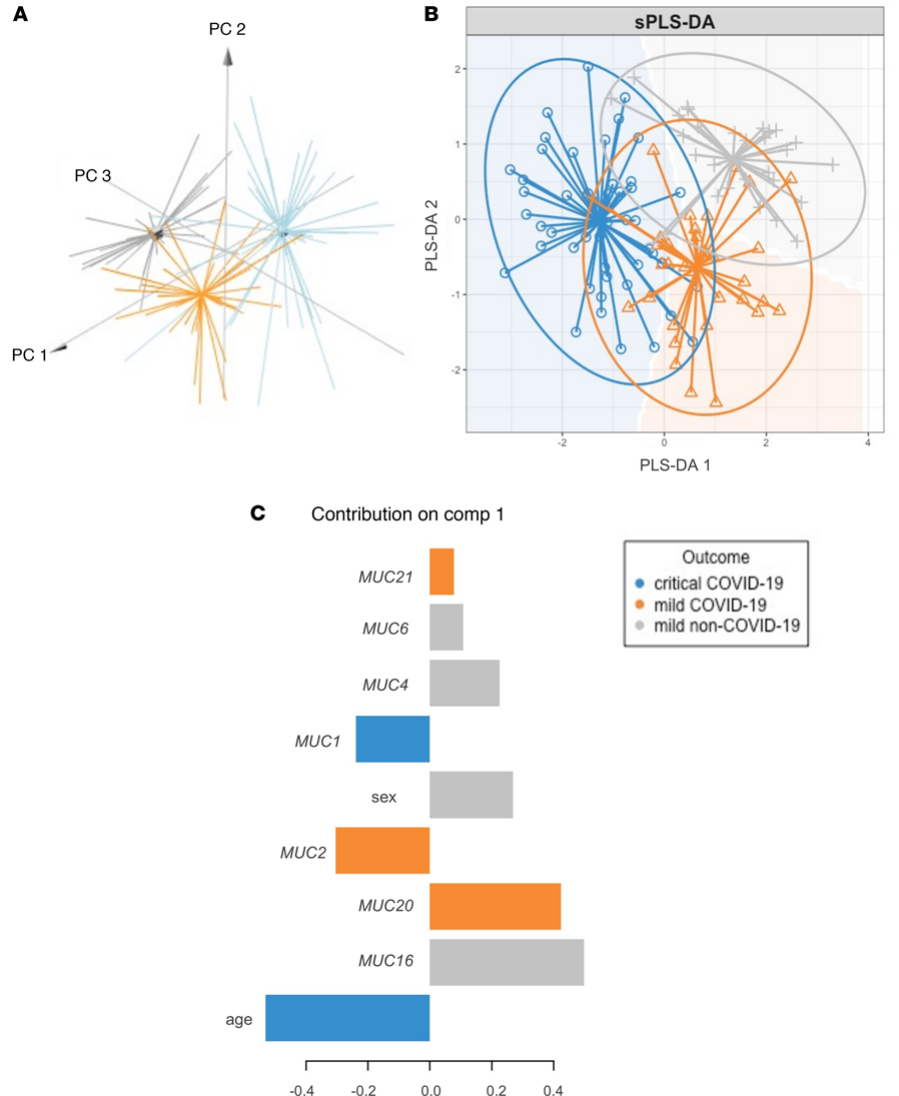
Peripheral mucin mRNA expression levels as discriminators for critical COVID-19, mild COVID-19, and mild non–COVID-19. (**A**) 3D PCA plot based on mucin mRNA expression values, age, and sex from critically ill COVID-19 patients (*n* = 50), mild COVID-19 patients (*n* = 35), and mild non–COVID-19 patients (*n* = 30). PC1 explains 28.7% of the variation; PC2 explains 17.8% of the variation. (**B**) sPLS-DA plot based on mucin mRNA expression values, age, and sex as major predictors for critical COVID-19 (*n* = 50), mild COVID-19 (*n* = 35), and mild non–COVID-19 (*n* = 30). (**C**) Contribution plot indicating the main predictor variables contributing to component 1 of the sPLS-DA plot that discriminate between critical COVID-19 (*n* = 50; i.e., age and *MUC1* mRNA expression), mild COVID-19 (*n* = 35; i.e., *MUC2*, *MUC20*, and *MUC21* mRNA expression), and mild non–COVID-19 (*n* = 30; i.e., *MUC4*, *MUC6*, and *MUC16* mRNA expression and sex) patients.

**Figure 5:**
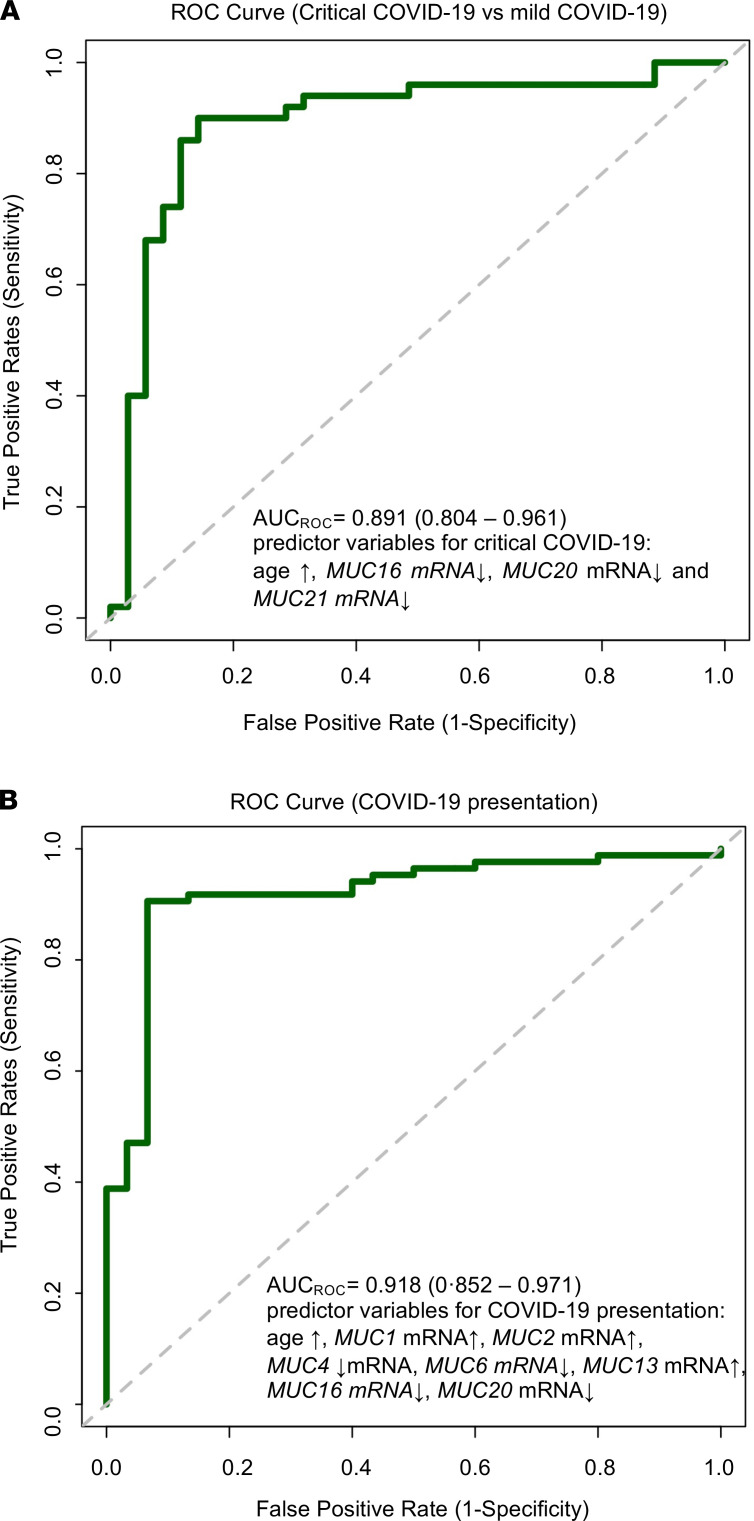
Peripheral mucin mRNA expression levels as variables associated with COVID-19 severity and presentation. (**A**) ROC curve and AUC for the prediction model of COVID-19 severity, i.e., critical COVID-19 (*n* = 50) or mild COVID-19 (*n* = 35) obtained by LASSO logistic regression. Included predictors were age and mRNA expression of *MUC16, MUC20,* and *MUC21*, with an AUC of 89.1% (95% CI: 80.4%–96.1%), a sensitivity of 90.0% (95% CI: 79.3%–96.2%), and a specificity of 85.7% (95% CI: 71.2%–94.6%). (**B**) ROC curve and AUC for the prediction model of COVID-19 presentation: COVID-19 positive (*n* = 85) or COVID-19 negative (*n* = 30) obtained by the LASSO logistic regression. Included predictors were age and mRNA expression of *MUC1, MUC2, MUC4, MUC6, MUC13, MUC16,* and *MUC20*, with an AUC of 91.8% (95% CI: 85.2%–97.1%), a sensitivity of 90.6% (95% CI: 83.0%–95.5%), and a specificity of 93.3% (95% CI: 79.7%–98.9%).

Subsequently, to test the hypothesis that patients with COVID-19 display an aberrant peripheral blood mucin mRNA signature, a principal component analysis (PCA) based on the mucin expression data was first undertaken. For the PCA and subsequent analyses described below, the log_2_ fold-change mucin expression data set was used because these values are more normally distributed and thus biologically more relevant. Furthermore, given that the different symptomatic patient groups were not age and sex matched, these 2 clinical parameters were also taken into account. Strikingly, mucin mRNA expression levels, age, and sex distinguished patients with critical COVID-19, mild COVID-19, and mild non–COVID-19 (Figure 4A), making them appropriate for further testing. To identify which of these variables are the major discriminators for critical COVID-19, mild COVID-19, or mild non–COVID-19, a sparse partial least-squares discriminant analysis (sPLS-DA) was then carried out. The sPLS-DA plot showed a clear discrimination among the different patient groups (Figure 4B), with *MUC1* mRNA expression and age as major determinants for critically ill COVID-19 patients, whereas expression of *MUC2*, *MUC20*, and *MUC21* mRNA were the best factors to identify patients with mild COVID-19 and sex, *MUC4*, *MUC6,* and *MUC16* mRNA expression to identify non–COVID-19 patients with mild Illness (Figure 4C). Thereafter, a least absolute shrinkage and selection operator (LASSO) regression with internal leave-one-out cross-validation and receiver operating characteristic (ROC) analysis identified age and mRNA expression of *MUC16*, *MUC20,* and *MUC21* as major variables associated with COVID-19 severity (i.e., critically ill or mild COVID-19), with an area under the ROC-curve (AUC_ROC_) of 89.1% and a sensitivity and specificity of 90.0% and 85.7%, respectively (Figure 5A and Supplemental Table 1). By using the same approach, we also showed that age and mRNA expression of *MUC1*, *MUC2*, *MUC4*, *MUC6*, *MUC13*, *MUC16*, and *MUC20* were the most accurate variables associated with the presence of COVID-19 among symptomatic patients (Figure 5B and Supplemental Table 2; AUC_ROC_ = 91.8%; sensitivity: 90.6%; specificity: 93.3%). Additionally, we performed an internal validation of our data set in which the 12 variables (mucins [*n* = 10], age, and sex) were included in a multinomial logistic regression model to classify the symptomatic patients in 1 of the 3 groups (i.e., critical COVID-19, mild COVID-19, and mild non–COVID-19). The data set was split into a 70:30 ratio where 70% (*n* = 80) was used for training the model and 30% (*n* = 35) for testing the model. The mean classification accuracy was 85% for the training set (multiclass AUC of 94.5%; Supplemental Table 3) and 80% for the test set (multiclass AUC of 93.21%; Supplemental Table 4). Remarkably, no patient with COVID-19 was misclassified as a non–COVID-19 patient in the independently validated test set. All above models clearly underline the potential usefulness of blood mucin mRNA expression levels for COVID-19 patient stratification.

**Figure 6:**
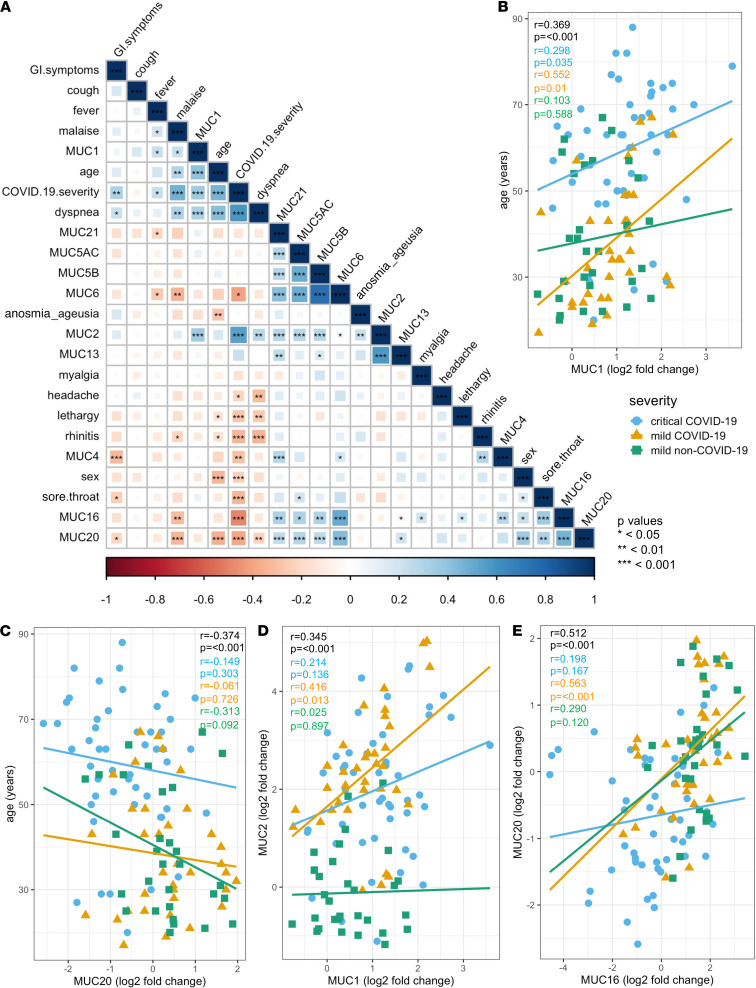
Associations of peripheral mucin mRNA expression with disease severity, age, sex, and symptoms. (**A**) Correlogram of the critically ill COVID-19 (*n* = 50), mild COVID-19 (*n* = 35), and mild non–COVID-19 (*n* = 30) patients. Spearman’s rank order correlation values (*r*) are shown from red (–1.0) to blue (1.0); *r* values are indicated by color and square size. *P* values are indicated by black asterisks (*<0.05; **<0.01; ***<0.001). (**B**) Correlation of *MUC1* mRNA expression with age. Statistics (*r* and *P* values) for the full data set (*n* = 135), critically ill COVID-19 patient data (*n* = 50), mild COVID-19 patient data (*n* = 35), and mild non–COVID-19 patient data (*n* = 30) are shown in black, blue, orange, and green, respectively. (**C**) Correlation of *MUC20* mRNA expression with age. Statistics (*r* and *P* values) for the full data set (*n* = 135), critically ill COVID-19 patient data (*n* = 50), mild COVID-19 patient data (*n* = 35), and mild non–COVID-19 patient data (*n* = 30) are shown in black, blue, orange, and green, respectively. (**D**) Correlation of *MUC1* mRNA expression with *MUC2* mRNA expression. Statistics (*r* and *P* values) for the full data set (*n* = 135), critically ill COVID-19 patient data (*n* = 50), mild COVID-19 patient data (*n* = 35), and mild non–COVID-19 patient data (*n* = 30) are shown in black, blue, orange, and green, respectively. (**E**) Correlation of *MUC16* mRNA expression with *MUC20* mRNA expression. Statistics (*r* and *P* values) for the full data set (*n* = 135), critically ill COVID-19 patient data (*n* = 50), mild COVID-19 patient data (*n* = 35), and mild non–COVID-19 patient data (*n* = 30) are shown in black, blue, orange, and green, respectively.

**Figure 7:**
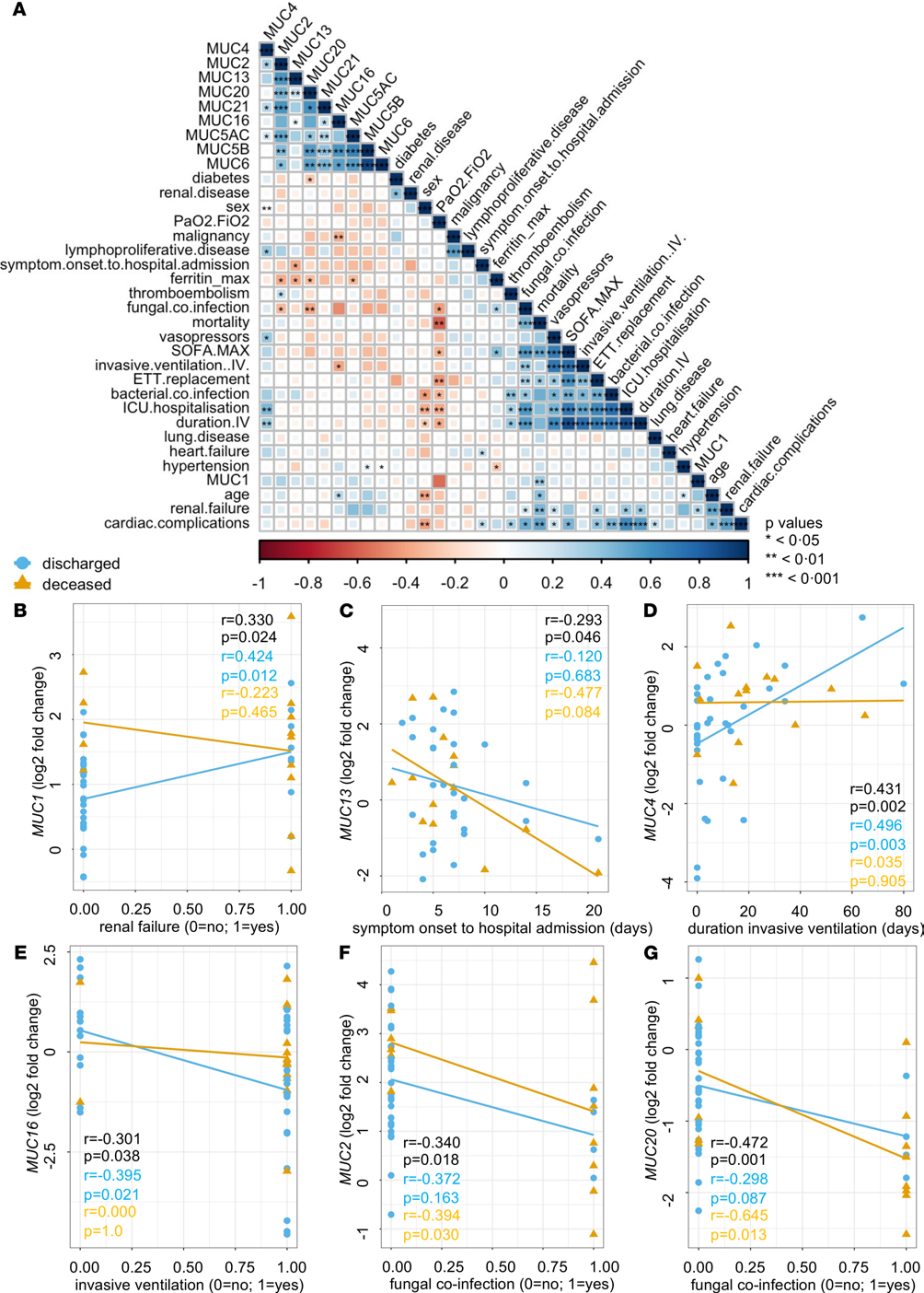
Associations of peripheral mucin mRNA expression with the clinical data of critically ill COVID-19 patients. (**A**) Correlogram of critically ill COVID-19 (*n* = 50) patients. Spearman’s rank order correlation values (*r*) are shown from red (–1.0) to blue (1.0); *r* values are indicated by color and square size. *P* values are indicated by black asterisks (*<0.05; **<0.01; ***<0.001). (**B**) Correlation of *MUC1* mRNA expression with renal failure. Statistics (*r* and *P* values) for the full data set (*n* = 50), discharged patient data (*n* = 36), and deceased patient data (*n* = 14) are shown in black, blue, and orange, respectively. (**C**) Correlation of *MUC13* mRNA expression with symptom onset to hospital admission. Statistics (*r* and *P* values) for the full data set (*n* = 50), discharged patient data (*n* = 36), and deceased patient data (*n* = 14) are shown in black, blue, and orange, respectively. (**D**) Correlation of *MUC4* mRNA expression with duration of invasive ventilation. Statistics (*r* and *P* values) for the full data set (*n* = 50), discharged patient data (*n* = 36), and deceased patient data (*n* = 14) are shown in black, blue, and orange, respectively. (**E**) Correlation of *MUC16* mRNA expression with the need for invasive ventilation. Statistics (*r* and *P* values) for the full data set (*n* = 50), discharged patient data (*n* = 36), and deceased patient data (n = 14) are shown in black, blue, and orange, respectively. (**F**) Correlation of *MUC2* mRNA expression with fungal coinfection. Statistics (*r* and *P* values) for the full data set (*n* = 50), discharged patient data (*n* = 36), and deceased patient data (*n* = 14) are shown in black, blue, and orange, respectively. (**G**) Correlation of *MUC20* mRNA expression with fungal coinfection. Statistics (*r* and *P* values) for the full data set (*n* = 50), discharged patient data (*n* = 36), and deceased patient data (*n* = 14) are shown in black, blue, and orange, respectively.

Furthermore, we also verified collinearity between the mucin mRNA expression data, disease severity (critical COVID-19, mild COVID-19, and mild non–COVID-19), and the clinical patient data (i.e., age, sex, symptoms) using Spearman’s correlation tests (Figure 6). *MUC1*, *MUC2*, *MUC4*, *MUC16,* and *MUC20* mRNA expression strongly correlated with COVID-19 severity, of which *MUC1* and *MUC20* mRNA expression also associated significantly with age (Figure 6, A–C) and *MUC16* and *MUC20* mRNA expression with sex (Figure 6A). A strong positive correlation was also seen between *MUC1* and *MUC2* mRNA expression and between *MUC16* and *MUC20* mRNA expression (Figure 6A) and specifically in the mild COVID-19 patient group (Figure 6, D and E). Significant relationships among other mucin mRNA expression profiles were also found (Figure 6A). Furthermore, a positive association of *MUC16* and *MUC20* mRNA expression with sore throat and *MUC1* and *MUC2* mRNA expression with dyspnea were found, whereas *MUC4* and *MUC20* mRNA expression associated negatively with gastrointestinal symptoms (Figure 6A). Finally, associations between mucin mRNA expression and the clinical characteristics of the critically ill COVID-19 patients were also investigated (Figure 7). Within the critically ill COVID-19 patient group, *MUC1* mRNA expression significantly correlated with mortality and renal failure (Figure 7, A and B), *MUC13* mRNA expression with the time between symptom onset and hospital admission (Figure 7, A and C), *MUC4* mRNA expression with the duration of invasive ventilation (Figure 7, A and D), and *MUC16* mRNA expression with the need for invasive ventilation (Figure 7, A and E). The association between *MUC1* mRNA expression and mortality is also shown in Figure 3A and Supplemental Figure 1A: high *MUC1* expression was mostly seen in deceased COVID-19 ICU patients (marked as filled blue bullets with a cross). In addition, a dominant MUC1 staining was also shown in lung tissue from deceased COVID-19 patients (Supplemental Figure 3, C and D) and in pulmonary biopsies collected from patients with an exudative phase of COVID-19 (Supplemental Figure 3, E and F), further underlining a potential role for MUC1 in the severe course of the disease. Furthermore, the occurrence of pulmonary fungal coinfection negatively correlated with *MUC2* and *MUC20* mRNA expression and specifically in the deceased patients (Figure 7, A, F, and G). As the occurrence of fungal coinfections associated with ferritin_max_ serum levels (Figure 7A), negative correlations were also identified for this clinical outcome parameter and *MUC2*, *MUC5AC*, *MUC13,* and *MUC20* mRNA expression (Figure 7A). Interestingly, only a few significant associations between mucin mRNA expression and the presence of a comorbidity were found, including *MUC4* mRNA expression with lymphoproliferative disease, *MUC5B,* and *MUC6* mRNA expression with hypertension, *MUC16* mRNA expression with malignancy, and *MUC20* mRNA expression with diabetes (Figure 7A). Given that these underlying diseases are present in a minority of the patients, this further suggests that most changes seen in mucin mRNA expression could be attributed to COVID-19. Of note, no significant correlations among obesity, autoimmune diseases, neurological complications, and sera IL-6 maximum levels with other clinical data and blood mucin mRNA expression levels were found.

### Remdesivir, favipiravir, baricitinib, and tocilizumab are able to reduce SARS-CoV-2–induced mucin expression, either directly or indirectly.

**Figure 8:**
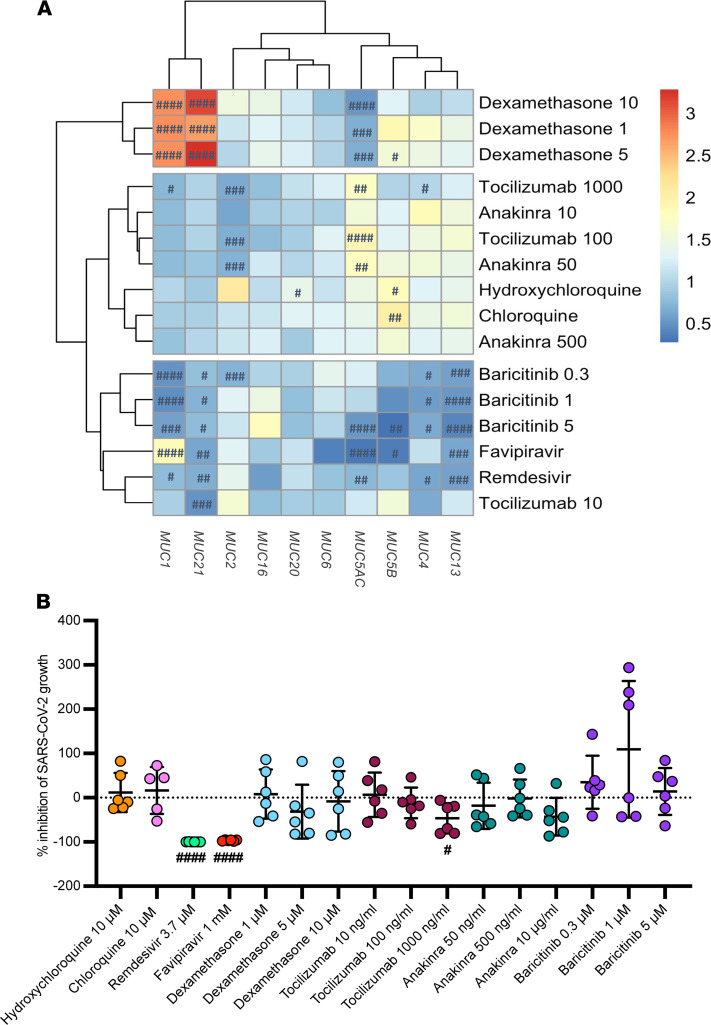
Ability of FDA-approved drugs for COVID-19 to reduce mucin overexpression in SARS-CoV-2–infected pulmonary epithelial cells. (**A**) Heatmap visualization of the relative mRNA expression data of *MUC1*, *MUC2*, *MUC4*, *MUC5AC*, *MUC5B*, *MUC13*, *MUC16*, *MUC20,* and *MUC21* in pulmonary (Calu3) epithelial cells infected with SARS-CoV-2 at 0.1 MOI for 2 hours and thereafter treated with a COVID-19 drug at different concentrations for 48 hours (*n* = 6). These include remdesivir (3.7 μM); favipiravir (1 mM); (hydroxy)chloroquine (10 μM); dexamethasone (1, 5, and 10 μM); tocilizumab (10, 100, and 1000 ng/mL); anakinra (50 and 500 ng/mL, 10 μg/mL), and baricitinib (0.3, 1, 5 μM). Significant differences between treated and untreated cells upon SARS-CoV-2 infection are indicated by ^#^*P* < 0.05; ^##^*P* < 0.01; ^###^*P* < 0·001; ^####^*P* < 0.0001. One-way ANOVA, Tukey’s post hoc multiple-comparison test. (**B**) Percentage of inhibition of SARS-CoV-2 growth in Calu3 cells upon treatment with a COVID-19 drug (*n* = 6). Data are presented as mean ± SEM. Significant differences between treated and untreated cells upon SARS-CoV-2 infection are indicated by ^#^*P* < 0.05; ^##^*P* < 0.01; ^###^*P* < 0·001; ^####^*P* < 0.0001. One-way ANOVA, Tukey’s post hoc multiple-comparison test.

We then investigated the ability of potential COVID-19 treatments to reduce aberrant mucin expression triggered by SARS-CoV-2, as shown in Supplemental Figure 4. Overall, in vitro stimulation of pulmonary epithelial Calu3 cells with several therapeutic drugs at certain doses showed that remdesivir and baricitinib were able to significantly reduce *MUC1*, *MUC2*, *MUC4*, *MUC5AC*, *MUC5B*, *MUC13,* and *MUC21* mRNA expression upon SARS-CoV-2 infection (Figure 8A and Supplemental Figure 4). Also, favipiravir (another antiviral agent) was able to decrease *MUC4*, *MUC5AC*, *MUC5B*, *MUC13,* and *MUC21* mRNA expression and to increase *MUC1* mRNA expression, whereas tocilizumab significantly reduced *MUC1*, *MUC2*, *MUC4,* and *MUC21* mRNA expression (Figure 8A and Supplemental Figure 4). On the contrary, *MUC1* and *MUC21* mRNA expression were significantly increased upon dexamethasone treatment (Figure 8A and Supplemental Figure 4). No significant alterations in *MUC16* and *MUC20* mRNA expression upon SARS-CoV-2 infection and treatment were found. When investigating the viral load in the supernatants of the cells after treatment, remdesivir and favipiravir and to a lesser extent tocilizumab (at a dose of 1000 ng/mL) had a significant impact on the growth of SARS-CoV-2, with almost complete inhibition of virus infection in epithelial cells upon remdesivir and favipiravir treatment (Figure 8B). This suggests an indirect effect of remdesivir and favipiravir on mucin expression through viral growth inhibition, whereas tocilizumab probably had an effect on both viral growth and mucin expression. On the contrary, as baricitinib was able to strongly reduce aberrant mucin expression upon infection comparable to the mucin expression seen in uninfected control cells, this therapeutic agent did not affect the viral growth. This highlights a direct impact of baricitinib on aberrant mucin expression.

## Discussion

In this study, we provide a comprehensive description of the transcriptional landscape of mucins in the peripheral blood of patients with COVID-19 with varying degrees of disease severity as potential determinants associated with COVID-19 presentation and severity.

The outcome of COVID-19 varies broadly; the majority of young, middle-aged individuals, particularly females, experience mild disease, whereas the risk for severe disease increases with age and mostly appears in the male population with an underlying medical condition (21, 22). Also, our study showed that men with a median age of 65 years were overrepresented in the critically ill COVID-19 patient group, which also included a minor subset of younger individuals without underlying disease, while mainly young to middle-aged females presented a mild disease course. This further underscores that men are more susceptible than women to the development of more severe disease. Along with their differential manifestation, patients with COVID-19 exhibit a wide variety of symptoms, and it is now clear that after COVID-19, long-lasting symptoms can also occur (22). All of this makes it difficult to predict disease progression and severity. Therefore, biomarkers that are able to predict which patients will develop a severe course of disease and thus should be treated as early as possible once infection occurs are urgently needed. Several studies have identified promising immune-based determinants for COVID-19 disease (17, 22–24) without focusing on mucins as critical components of innate immunity and acting as a first line of defense against pathogens (6, 20). Mucins play an important role in infectious disease progression in which expression of these glycoproteins is regulated by cytokines (8). Moreover, many viruses utilize these glycoproteins to enter the cells (20). Given that mucins are shed from SARS-CoV-2–infected respiratory epithelial cells (10), as shown in our study (Figure 8B and Supplemental Figures 2 and 4), and can enter the circulation via mucosal barrier injury (10), establishing blood mucin expression profiles associated with disease presentation and severity and response to therapy will hold great potential to battle this COVID-19 pandemic. In our study, LASSO regression modeling determined a dynamic blood mucin mRNA signature that clearly distinguished patients with symptomatic COVID-19 from symptomatic non–COVID-19 patients based on expression of *MUC1*, *MUC2*, *MUC4*, *MUC6*, *MUC13*, *MUC16,* and *MUC20* and distinguished between mild and critically ill COVID-19 patients based on expression of *MUC16, MUC20,* and *MUC21* with high sensitivity and specificity for both models. It is interesting to note that *MUC21* mRNA expression was only selected as a variable associated with COVID-19 severity, whereas *MUC13*, *MUC4,* and *MUC6* mRNA expression were uniquely identified as variables indicative for COVID-19 presentation. This highlights their potential in defining disease severity (mild or critical) or presence of COVID-19, as proven by the results from our sPLS-DA as well (Figure 4C). *MUC1* mRNA expression was, in addition to its association with COVID-19 presentation, identified as a major discriminant for critically ill COVID-19 patients, which is in line with what has previously been reported (25). Also, age was a predictor variable for COVID-19 presentation and severity, further underscoring that age is a well-known risk factor (22), which could probably drive the differences in mucin expression. However, the healthy control group, covering a wide age range (38–83 years), did not show major variability in mucin gene expression, as shown in Figure 3, suggesting a normal epithelial barrier function even in the aged, uninfected healthy population. Because we did not include aged, critically ill non–COVID-19 controls in our study, it remains to be further elucidated whether age could affect mucin expression upon inflammation.

Moreover, striking correlations also existed among the different key mucins for COVID-19 presentation and severity and between these mucins and certain clinical parameters. Of particular interest is the strong correlation between mortality, renal failure, and *MUC1* mRNA expression and between mechanical ventilation and *MUC4* and *MUC16* mRNA expression. It is noteworthy that in autosomal dominant tubulointerstitial kidney disease, renal failure is also associated with aberrant MUC1 expression (26). Expression of several mucins is also negatively correlated with the highest ferritin levels found in the sera and the presence of fungal coinfection, more specifically an *Aspergillus fumigatus* infection, during ICU stays. Mucins have the ability to absorb iron (27), which could possibly explain the inverted association between mucin and ferritin levels seen in critically ill COVID-19 patients. Ferritin levels in the serum are in turn a risk factor for fungal secondary infections (28). *Aspergillus* infections have recently been proposed to be included in the outcome parameters for COVID-19 given that one third of COVID-19 patients admitted to the ICU developed pulmonary aspergillosis, resulting in higher mortality (29), as was also shown in our study (Figure 7A). Because aspergillus disease manifestations are more heterogeneous in patients with COVID-19, these mucin and ferritin levels could potentially be additional parameters for distinguishing between airway colonization and invasive disease and for starting appropriate antifungal therapy (29). Finally, significant correlations were also found between *MUC16* and *MUC20* mRNA expression and the presence of sore throat, a symptom only found in the mild ambulatory patient groups, between *MUC1* and *MUC2* mRNA expression and dyspnea, and between *MUC4* and *MUC20* mRNA expression and gastrointestinal symptoms. The latter 2 symptoms are predominantly found in critically ill COVID-19 patients. All these correlations further emphasize the potential role of peripheral mucin mRNA expression levels in shaping the course of COVID-19.

Since no specific antiviral drugs for the treatment of human coronavirus infections are currently available, the urgent need for an effective treatment has led to clinical trials using repurposed drugs without preclinical evidence in advance of their efficacy against SARS-CoV-2 infection (30). Suppressing the replication of SARS-CoV-2 as well as the host factors mediating the outcome of the disease could be beneficial in preventing or alleviating disease. In cell culture and animal models, several repurposed drugs have already shown their potential to inhibit SARS-CoV-2 replication (30–32). In our study, we further investigated the efficacy of repurposed COVID-19 drugs to reduce mucin hyperexpression in SARS-CoV-2–infected epithelial cells, and in particular the mucins defining the signature associated with COVID-19 presentation and severity. Overall, baricitinib, tocilizumab, favipiravir, and remdesivir were able to significantly alter mucin expression, albeit directly or indirectly via inhibition of viral replication. Baricitinib, a selective inhibitor of JAK 1 and JAK 2, inhibits the intracellular signaling pathways of cytokines, such as IL-1β, IL-6, and TNF-α (33), and as proven in our study, baricitinib inhibits mucin signaling, given that expression of these glycoproteins is regulated by these cytokines during infection and inflammation (8). Although several clinical trials showed no clinical effect of tocilizumab on the outcome of patients with severe COVID-19 (34), this IL-6R inhibitor was able to reduce both mucin expression and viral replication. This finding further underlines the recent suggestion of tocilizumab as a useful treatment strategy to prevent the evolution of COVID-19 from a mild-moderate to severe state (34). Remdesivir and favipiravir, two antiviral agents blocking RdRp, inhibited mucin expression indirectly via complete inhibition of SARS-CoV-2 replication. The strong antiviral activity of favipiravir was also confirmed in an animal model for SARS-CoV-2 (30), and clinical studies are currently ongoing. Despite the benefits of remdesivir, substantial morbidity and mortality due to COVID-19 remain (35). Therefore, treatment should be given early in infection and before overreaction of the host immune response (i.e., the cytokine storm) occurs. Furthermore, it was recently shown that the combination of baricitinib plus remdesivir was superior to remdesivir alone (35), further highlighting the great potential of both drugs to treat COVID-19. In addition, dexamethasone is used to treat severe COVID-19 because it might significantly reduce the mortality rate of COVID-19 patients receiving respiratory support (32). Here, we showed that this drug increases mucin expression, and in particular expression of *MUC1*, a major determinant associated with critical disease. Given that MUC1 can serve as an antiinflammatory factor in the airways later in the disease course (36), its role in severe COVID-19 remains to be further investigated. On the contrary, chloroquine and hydroxychloroquine did not affect mucin expression nor viral replication. This underlines the lack of scientific basis to use the latter drugs for COVID-19, as suggested before (30). At this point, additional clinical studies are required to further explore the ideal combination of drugs able to reduce both viral replication and mucin expression at disease onset as well as the time frame of treatment options when infection occurs.

Our study has some limitations. First, we were unable to 1) concurrently enroll a critically ill age-matched non–COVID-19 control group at our ICU to define the peripheral mucin mRNA signature in this patient group and to 2) collect mucus samples from non–COVID-19 ICU patients receiving invasive ventilation to calculate which mucins were differentially expressed in the lungs specifically due to COVID-19. A decline in hospital presentation for diseases other than COVID-19 hampered the collection of samples for an adequately matched critical illness control group. Second, it remains to be further substantiated whether age has an impact on peripheral mucin expression upon severe inflammation independent of COVID-19 and whether mucin expression levels are considerably different between patients with critical COVID-19 and acute respiratory diseases arising from other pathologies. This has already been acknowledged for MUC1, where increased levels of this mucin have also been described in patients with ARDS and sepsis-induced acute lung injury (37, 38) but remains unclear for the other mucins investigated in this study. Finally, the blood samples from the critically ill COVID-19 cohort were collected upon admission, and several clinical parameters were recorded during the ICU stay. Investigating mucin expression in the peripheral blood during the course of the hospital stay would therefore be an added value to further strengthen the correlations found in this study between mucin expression levels and clinical outcome parameters and to unravel their causal relationship.

Nevertheless, the strength of our study lies in a multifaceted blood mucin mRNA signature that has the ability to identify symptomatic patients with COVID-19 and discriminate between mild and critical disease. Also, measuring expression of several mucins resulted in a good classification of patients in the different patient groups with high accuracy, underlining the importance of investigating a panel of several mucins rather than measuring single mucin expression for COVID-19 patient stratification. Furthermore, this peripheral mucin mRNA signature also has the potential to collectively and individually guide treatment options and provides a basis for future research in investigating many clinical and basic research questions. These include 1) whether specific mucin traits reflect breaches in the respiratory and gastrointestinal and urogenital tracts since associations of mucin with gastrointestinal symptoms and renal failure were also found; 2) whether these mucin changes are causes or consequences of disease progression; 3) what the cell source is of the mucin mRNA detected in the blood, given that some mucins can also be expressed by immune cells besides epithelial cells of different organ systems (13, 18, 19), which will provide insights into the causal role of mucins in SARS-CoV-2 pathogenesis; and 4) which mucin mRNA isoforms are associated with COVID-19 severity, allowing identification of high-risk and low-risk patients. Mucins are highly polymorphic, and the presence of genetic differences can alter gene expression, resulting in several mRNA isoforms via alternative splicing. While most isoforms encode similar biological functions, some alter protein function, resulting in progression toward disease (39).

In summary, we identified this dynamic mucin mRNA signature as having great potential to improve COVID-19 patient stratification, thereby diminishing the life-threatening potential of SARS-CoV-2 infection. Furthermore, an adequate independent external validation (i.e., mucin mRNA measurements at baseline, during infection, and after infection) in other COVID-19 and concurrent non–COVID-19 age-matched cohorts is recommended to elucidate the prognostic/predictive potential of this peripheral mucin mRNA signature for COVID-19.

## Methods

### Study design and participants.

Between August 20, 2020, and April 6, 2021, 50 critically ill COVID-19 patients hospitalized at the tertiary ICU of the Antwerp University Hospital, Belgium, and 35 ambulatory COVID-19 patients with no or mild-moderate symptoms were enrolled for this study (Table 1). The severity of the disease was classified in line with the WHO scale as (i) mild; (ii) moderate (symptoms such as fever, cough, dyspnea, but no signs of severe pneumonia); (iii) severe: clinical signs of pneumonia plus the need for respiratory support (high-flow oxygen and/or mechanical ventilation); (iv) critical: presence of ARDS (40) and/or sepsis or multiple organ failure (septic shock) (http://www.who.int/blueprint/priority-diseases/key-action/novel-coronavirus/en/).

Ambulatory patients with mild common cold symptoms (*n* = 30, Table 1) and healthy controls (*n* = 20, Table 1), which were all negative for COVID-19 as confirmed by viral PCR, were included as control groups. The ambulatory COVID-19–positive and COVID-19–negative patient groups and healthy controls were recruited at 5 general practitioner practices and 1 triage station in Antwerp, Belgium.

The recorded data for the ICU patients, all presenting with hallmarks of ARDS, the most severe form of lung injury (40), included 1) demographic and anthropometric data; 2) several markers of severity of pulmonary involvement (i.e., necessity for invasive ventilation, duration of invasive ventilation, unforeseen replacement of ETTs due to mucus impaction, lowest P_a_O_2_/F_i_O_2_ ratio during ICU stay); 3) assessment of severity of disease, i.e., duration of hospitalization, renal failure necessitating dialysis, occurrence of secondary bacterial or fungal infection during ICU stay, routine measurements of IL-6 (pg/mL; Elecsys IL-6 assay, Roche) and ferritin (μg/l; Atellica IM Fer assay, Siemens Healthineers) serum levels at admission onset and their maximum serum levels during ICU stay, cardiac/neurological/thromboembolic complications, the highest SOFA score (41), and in-hospital mortality. These data were retrieved from the patient data management system (Metavision, IMD software) and are summarized in Table 1.

Blood sampling for unraveling the peripheral blood mucin mRNA landscape was performed upon admission at the ICU for the critically ill COVID-19 patients or, in case of the ambulatory patients and healthy controls, at the same time as their COVID-19 PCR test in order to recruit COVID-19–positive and COVID-19–negative patients. Additionally, ETTs from mechanically ventilated ICU patients with COVID-19 were collected upon ETT replacement due to mucus obstruction (*n* = 13) as well as lung tissue collected as a part of volume reduction surgery for diffuse emphysema in ICU patients with the exudative phase of COVID-19 (*n* = 2) and from deceased ICU patients with COVID-19 upon autopsy (*n* = 2).

All blood and endotracheal mucus samples were immediately stored in PAXgene RNA blood tubes (PreAnalytiX) and TRIzol reagent (Thermo Fisher Scientific), respectively, at –80°C until RNA extraction and subsequent mucin gene expression analyses. Postmortem lung tissue was embedded in paraffin for subsequent mucin immunohistochemical staining.

### SARS-CoV-2 growth and biosafety.

The SARS-CoV-2 isolate 2019-nCoV/Italy-INMI1, available at the European Virus Archive-Global (EVAg) database, was used for the cell culture experiments. SARS-CoV-2 was subjected to passages in Vero cells (green monkey kidney; ATCC CCL-81), grown in EMEM (Lonza) supplemented with 2 mM L-glutamine, 100 U/mL to 100 μg/mL of penicillin-streptomycin and 2% heat-inactivated FBS, before usage in the cell culture experiments. The infectious viral titers in the cell-free supernatant were determined by a standard TCID50 titration on Vero cells. All experiments entailing live SARS-CoV-2 were conducted in the biosafety level 3 facility at the Institute for Tropical Medicine, Antwerp, Belgium.

### Screening the ability of different therapies for COVID-19 to reduce mucin hypersecretion.

Calu3 (lung adenocarcinoma ATCC HBT-55) cells were grown in MEM (Gibco) supplemented with 10% heat-inactivated FCS, 100 U/mL penicillin, 100 μg/mL streptomycin, 1× MEM nonessential amino acids, and 1 mM sodium pyruvate. For viral infection, cells were seeded in 24-well plates at a concentration of 5 × 10^5^ cells/mL. After reaching confluence, the cells were inoculated with SARS-CoV-2 at MOI of 0.1 for 2 hours, thereafter washed and treated with an FDA-approved drug for COVID-19 at different concentrations for 48 hours. The concentrations were selected based on previously published experiments (30, 31, 42–46). These included remdesivir (antiviral; 3·7 μM, Bio-Connect BV; ref. 31); favipiravir (antiviral; 1 mM, Bio-Connect BV; ref. 30), (hydroxy)chloroquine (antimalarial drug; 10 μM, Sigma-Aldrich; ref. 31); dexamethasone (corticosteroid; 1 μM, 5 μM, 10 μM, Sigma-Aldrich; ref. 45); tocilizumab (anti–IL-6R; 10 ng/mL, 100 ng/mL, 1000 ng/mL, Bio-Connect BV; ref. 44); anakinra (anti–IL-1; 50 ng/mL, 500 ng/mL, 10 μg/mL, Sigma-Aldrich; ref. 42); and baricitinib (JAK1/2 inhibitor; 0.3 μM, 1 μM, 5 μM, Bio-Connect BV; refs. 43, 46). Untreated SARS-CoV-2–infected cells were included as a control group. Forty-eight hours after treatment, cells and supernatants were lysed for RNA extraction to quantify mucin mRNA expression and viral load by RT-PCR. The cell culture experiments were performed containing 6 technical replicates for each treatment concentration.

### RNA isolation and quality control.

Total RNA was extracted from the collected blood samples using the PAXgene RNA blood kit (PreAnalytiX), from the endotracheal mucus samples using the RNeasy midi plus kit (Qiagen), from the lysed cells using the Nucleospin RNA plus kit (Macherey-Nagel), and from the cell supernatants using the QIAmp Viral RNA mini kit (Qiagen), following the manufacturer’s instructions. The concentration and purity of the RNA were evaluated using the NanoDrop ND-1000 UV-Vis Spectrophotometer (Thermo Fisher Scientific) and Qubit Fluorometer (Qubit Broad Range RNA kit, Thermo Fisher Scientific).

### Mucin mRNA expression by RT-PCR.

One μg RNA was converted to cDNA by reverse transcription using the SensiFast cDNA synthesis kit (Bioline). Relative mucin gene expression was then determined by SYBR Green RT-qPCR using the GoTaq qPCR master mix (Promega) on a QuantStudio 3 Real-Time PCR instrument (Thermo Fisher Scientific). Standard validated QuantiTect primers available from Qiagen were used for *GAPDH* (QT00079247), *ACTB* (QT00095431), *MUC1* (QT00015379), *MUC2* (QT01004675), *MUC4* (QT00045479), *MUC5AC* (QT00088991), *MUC5B* (QT01322818), *MUC6* (QT00237839), *MUC13* (QT00002478), *MUC16* (QT01192996), *MUC20* (QT00012068), and *MUC21* (QT01159060). All RT-PCR reactions were performed in duplicate and involved an initial DNA polymerase activation step for 2 minutes at 95°C, followed by 40 cycles of denaturation at 95°C for 15 seconds and annealing/extension for 1 minute at 60°C. A no template control (NTC) and a no amplification control (NAC) were included as negative controls in the RT-PCR experiments. The PCR efficiency for each primer pair and the obtained amplicon were validated from standard melt curves, and if samples were run on more than one 96-well plate, a sample served as interplate calibrator. Quality control and stability of the housekeeping genes *ACTB* and *GAPDH* were performed using qbase+ software (Biogazelle). Relative mRNA expression of the mucin genes was normalized to the expression of *ACTB* and *GAPDH* using qbase+ software (Biogazelle). In the blood, mucin mRNA expression values were also expressed as log_2_-transformed fold-changes normalized to the mucin expression levels in healthy controls using the ΔCt method as described before (47). The following formula was used: 2[–(ΔCt (sample from symptomatic patient group (i.e. critically COVID-19, mild COVID-19, or mild non–COVID-19)) – ΔCt (pooled samples from healthy control group))] of which ΔCt = Ct (mucin gene) – Ct (mean of *ACTB* and *GAPDH*).

### SARS-CoV-2 RT-PCR.

To measure viral RNA in the supernatants of the treated and nontreated SARS-CoV-2–infected cells, the sensiFAST Probe No-Rox One-Step kit (Bio-Rad) was used on a Quantstudio PCR platform (Thermo Fisher Scientific). A 25 μL reaction contained 5 μL RNA, 12.5 μL of 2 × SensiFAST mix provided with the kit, 0.25 μL RT, 0.5 μL RiboSafe RNAse inhibitor, 0.4 μL forward primer (25 μM), 0.4 μL reverse primer (25 μM), 0.5 μL probe (10 μM) targeting the SARS-CoV-2 E gene, and 3.95 μL H_2_O. We incubated the reactions at 45°C for 10 minutes for reverse transcription, 95°C for 5 minutes for denaturation, followed by 45 cycles of 95°C for 5 seconds and 58°C for 20 seconds. Analysis was performed using Quantstudio5 Design & Analysis software and qbase+ software to determine Ct and measure the percentage of growth inhibition.

### Immunohistochemistry.

Immunohistochemistry with MUC1 was performed on formalin-fixed paraffin-embedded human lung tissue from either autopsied COVID-19 patients or from biopsied lung tissue from patients with COVID-19. Lung tissue collected as paired control tissue of patients with lung cancer served as the control. Briefly, tissue sections of 5 μm thickness were mounted on glass slides (Superfrost, Thermo Fisher Scientific), deparaffinized, and boiled in citrate buffer (pH 6) and incubated overnight with anti–Muc-1 antibody (sheep pAb, AF6298, R&D Systems, 1:500) at 4°C. Primary antibody was detected by biotinylated donkey anti-sheep antibody (Jackson ImmunoResearch, 1:200) combined with HRP-conjugated Extravidin (Sigma-Aldrich) and developed with 5′, 5′ diaminobenzidine (DAKO, Agilent Technologies). Slides were counterstained with hematoxylin and visualized by Zeiss Scope A1 as described previously (48).

### Statistics.

According to the Strengthening the Reporting of Observational Studies in Epidemiology (STROBE) (49), patient characteristics are expressed as median (IQR) for continuous variables and analyzed by the 2-way ANOVA test (GraphPad Prism 9.0.0 software), whereas differences between proportions indicated as *n* (%) were analyzed by Pearson χ^2^ test (IBM SPSS Statistics 27).

Significant differences in (a) mucin mRNA expression between patients with mild and critical COVID-19, non–COVID-19 patients with mild disease and healthy controls, SARS-CoV-2–infected and uninfected pulmonary cells, and treated and untreated SARS-CoV-2–infected cells and (b) inhibition of SARS-CoV-2 growth in treated and untreated pulmonary cells were determined by the ANOVA test (GraphPad Prism), and data are presented as means ± SEM. Significance levels are indicated on the graphs and were corrected for multiple testing using the Tukey-Kramer and Dunn’s post hoc multiple-comparison tests.

PCA (unsupervised method) and sPLS-DA (supervised method) were performed to determine COVID-19 severity based on a set of predictor variables (i.e., the peripheral mRNA expression levels of mucins, age, and sex). PCA was carried out using the R (v3.6.1) packages pca3d (v0.10.2), rgl (v0.106.8), Factoextra (v1.0.7), FactoMineR (v2.3), and devtools (v2.4.1) in Rstudio (1.1.456), whereas sPLS-DA was done using the Github package Mixomics with 12 variables in the first component. Subsequently, a LASSO regression with leave-one-out cross-validation and ROC analysis was also carried out to investigate which variables (peripheral mucin expression levels, age, and sex) are the most accurate predictors for COVID-19 severity and presentation. These analyses were performed using the R packages glmnet (v4.1-1), gplots (v3.1.1), ROCR (v1.0-11), foreign (v0.8-81), and propCIs (v0.3-0) in Rstudio. In addition, multinomial logistic regression was performed on the variables (peripheral mucin expression levels, age, and sex) to internally classify and predict COVID-19 and non–COVID-19 cases. To investigate the quality of prediction, the multiclass AUC was also examined. The following R packages were used: dplyr (version 1.0.5), nnet (version 7.3-16), and pROC (version 1.17.0.1). Finally, Spearman’s correlations between peripheral mucin mRNA expression and the clinical data (i.e., COVID-19 severity, age, sex, and symptoms) were identified among the different patient cohorts and between mucin mRNA expression and the clinical patient data separately in the critically ill COVID-19 group using R in Rstudio. Correlograms plotting the Spearman’s rank correlation coefficient (*r*) between all parameter pairs were created (corrplot v0.84 package) as well as correlation plots displaying specific correlations of mucin mRNA expression and clinical parameters (ggplot2 v3.1.1 package). *P* values were calculated using corr.test (psych v1.8.12) and graphed (ggplot2 v3.1.1). *P* values of less than 0.05 were considered significant. R code to reproduce the figures is available at https://github.com/AnnemiekeSmet/MUCOV_analysis.git

### Study approval.

This study was approved by the Ethical Committee of the Antwerp University Hospital (20/14/176 [B3002020000059] and 20/43/555 [B3002020000193]), and written informed consent was obtained from the healthy controls; the patients; or in the case of intubated ICU patients, by their closest relative. Samples were registered and stored until analysis in the Biobank Antwerpen, Antwerp, Belgium (BE 71030031000; BBMR-ERIC, Belgian no. access: 1, Last: April 10, 2021. [BIORESOURCE]).

## Author contributions

AS and BYDW conceived, designed, and supervised the study. AS wrote the first draft of the manuscript, analyzed the raw data, interpreted the obtained results, performed the statistical analyses, and designed the graphs. TB, WA, JM, A Hauner, and LH contributed to the RT-PCR assays and cell culture experiments. KL provided intellectual input for the statistical analysis of the mucin expression data. JDM, SMK, and SKS provided intellectual support in the study design. KKA supervised the virus culture experiments. PJ, BL, AV, RJ, KD, and MH supervised the inclusion of the blood samples from the ICU COVID-19 patients, and PJ, RJ, and KD provided intellectual support regarding the clinical ICU data. A Hotterbeekx contributed to the immunohistochemical stainings. SC and VH supervised the inclusion of the blood samples from the ambulatory patients at the general practices and triage center. All authors discussed the results and commented on the manuscript.

## Supplementary Material

Supplemental data

ICMJE disclosure forms

## Figures and Tables

**Table 1:**
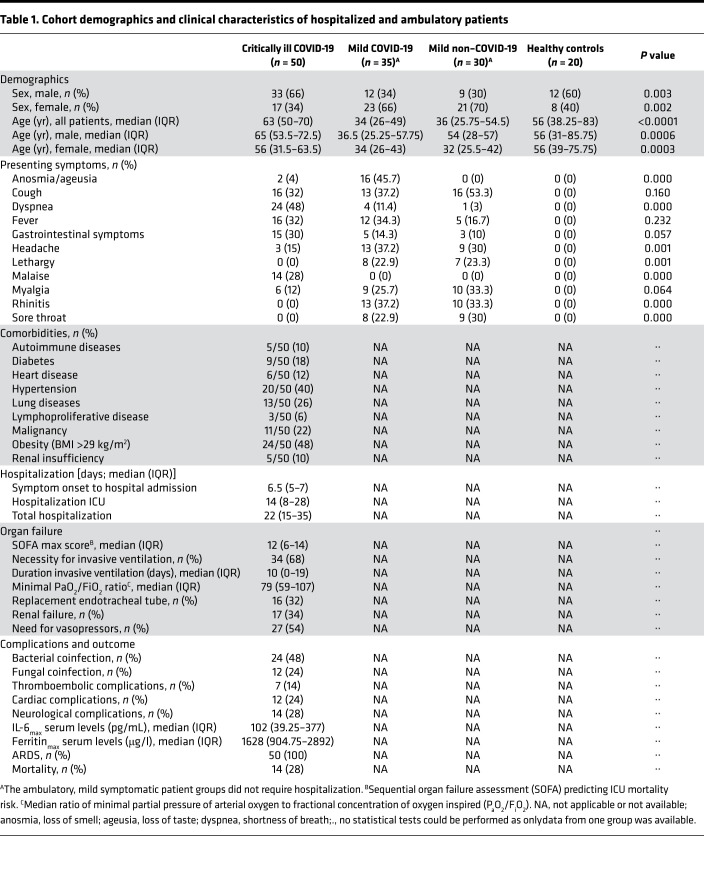
Cohort demographics and clinical characteristics of hospitalized and ambulatory patients

## Version 1 08/27/2021

In-Press Preview

## Version 2 10/08/2021

Electronic publication
