# Supplementary material for: A dynamic mucin mRNA signature associates with COVID-19 disease presentation and severity
Source: JCI Insight. 2021 Oct 8;6(19):e151777. doi: 10.1172/jci.insight.151777 (PMC8525642; doi:10.1172/jci.insight.151777)
Supplement: Supplemental data [file jciinsight-6-151777-s080.pdf]

Supplementary Material

A dynamic mucin mRNA signature associates with COVID-19 disease presentation and severity

Annemieke Smet[1,2,*], Tom Breugelmans[1,2], Johan Michiels[3], Kevin Lamote[1,2,4], Wout Arras[1,2], Joris G. De Man[1,2], Leo Heyndrickx[3], Anne Hauner[3], Manon Huizing[5], Surbhi Malhotra-Kumar[6], Martin Lammens[7], An Hotterbeekx[8], Samir Kumar-Singh[8], Aline Verstraeten[9], Bart Loeys[9], Veronique Verhoeven[10], Rita Jacobs[1,2,11], Karolien Dams[1,2,11], Samuel Coenen[10], Kevin K. Ariën[3,12], Philippe G. Jorens[1,2,11] & Benedicte Y. De Winter[1,2,13]

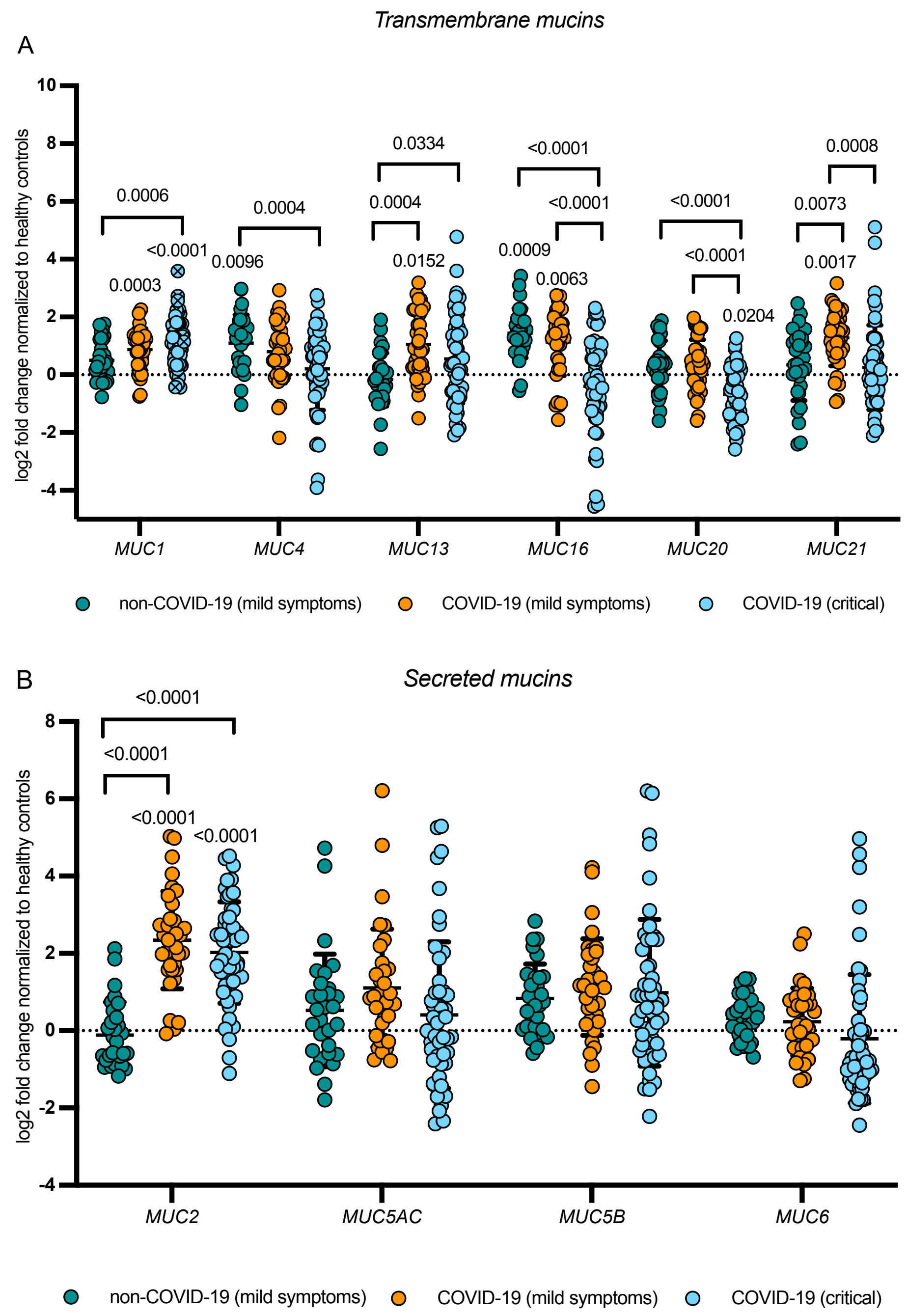

Supplementary Figure 1. Transmembrane (A) and secreted (B) mucin mRNA expression in the blood of patients with critical COVID-19 (n=50), mild COVID-19 (n=35) and mild non-COVID-19 (n=30) is shown in a dot plot as $\log_2$ fold changes compared to healthy controls (n=20). Each filled circle represents a patient and a blue filled circle with a cross, as shown for MUC1, denoted deceased patients in the critically ill COVID-19 group. Significant differences between critically ill COVID-19, mild COVID-19 or mild non-COVID-19 patients and healthy controls as well as among the different symptomatic patient groups are indicated by a p-value. One-Way ANOVA, Tukey's post-hoc multiple comparison test. All values are reported as mean ± SEM.

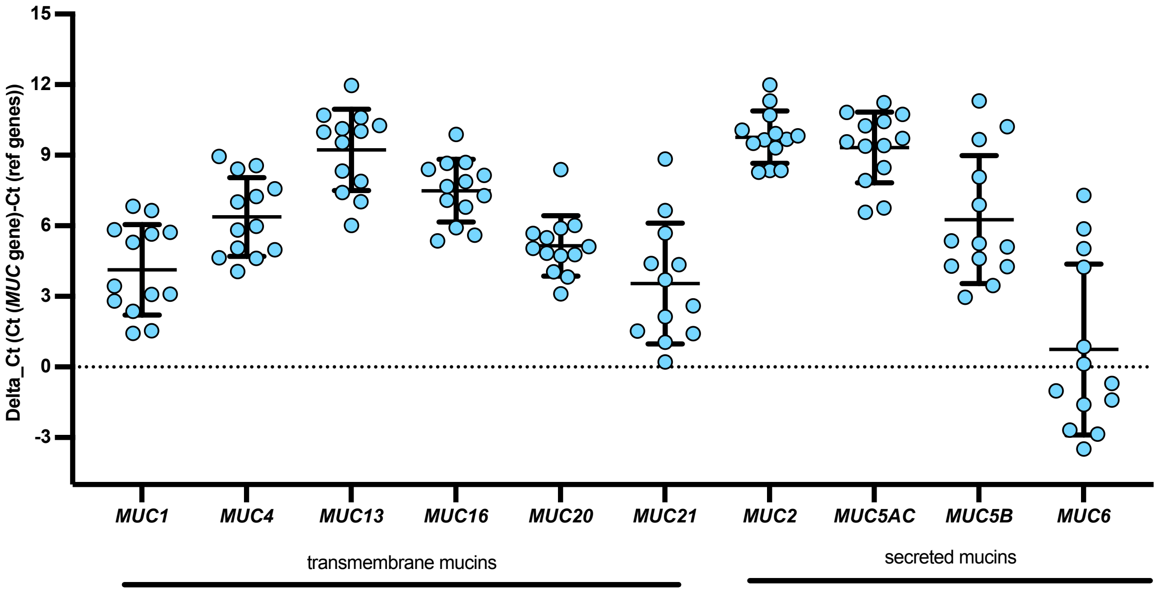

Supplementary Figure 2. Presence of transmembrane and secreted mucins in mucus samples from critically ill COVID-19 patients that are mechanically ventilated due to airway obstruction caused by mucus hypersecretion (n=13). The mRNA expression levels, obtained by RT-PCR, are expressed here as delta_Ct values, i.e.: the difference between the Ct value of the *MUC* gene of interest and the average Ct value of the housekeeping genes (*B-actin*, *GAPDH*). The lower the delta_Ct value, the higher the mucin mRNA expression level. A clear variation in respiratory mucin expression among the different COVID-19 patients is noted.

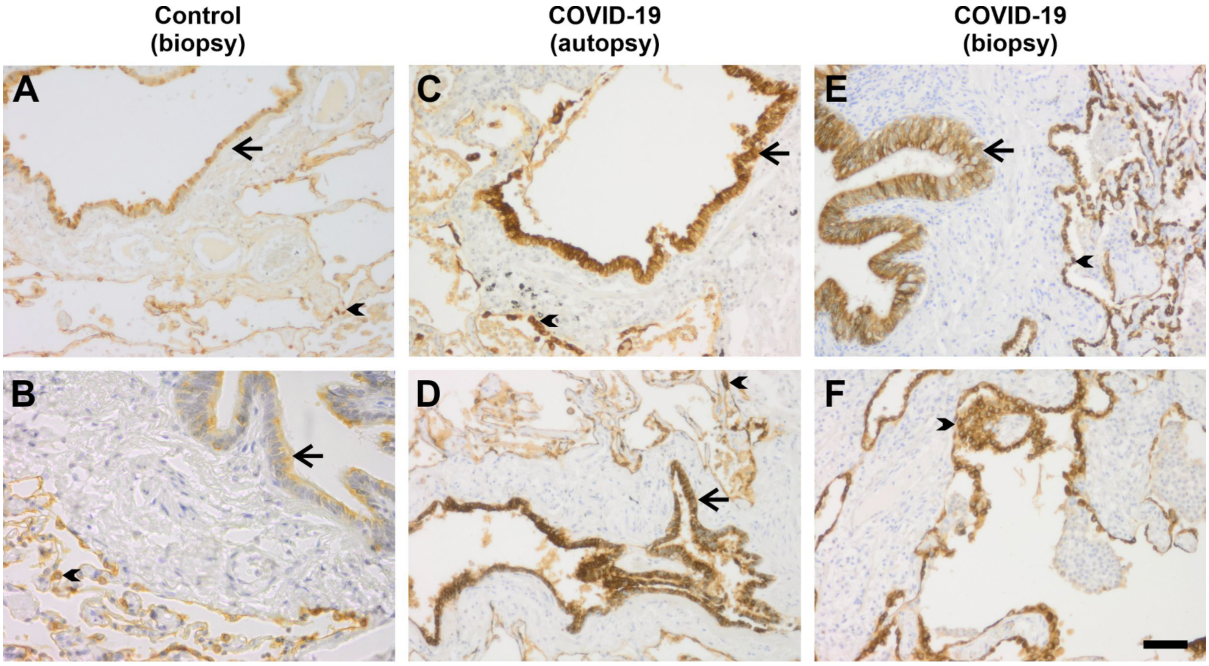

Supplementary Figure 3. MUC1 immunostaining in lungs of COVID-19 patients. (A, B) Lung tissue collected as paired control tissue of patients with lung cancer (n=2) showing normal localization of MUC1 staining in the bronchiolar epithelia (arrow) and in type-II pneumocytes (arrowheads). (C, D) Autopsied lungs of COVID-19 patients (n=2) with intense staining of MUC1 both in airway epithelia (arrow) and type-II pneumocytes of regenerating alveoli where most of the alveolar structure was lost in patients dying of respiratory failure. D represents a terminal portion of the respiratory tree with coalescing masses of alveolar type-II pneumocytes. (E, F) Intense MUC1 immunostaining is also present in pulmonary resection specimens collected as a part of volume reduction surgery for diffuse emphysema in patients with an exudative phase of COVID-19 (n=2), suggesting that MUC1 upregulation is a part of early phases of COVID-19. Scale bar 20 µm. Each photomicrograph is from an independent patient/individual.

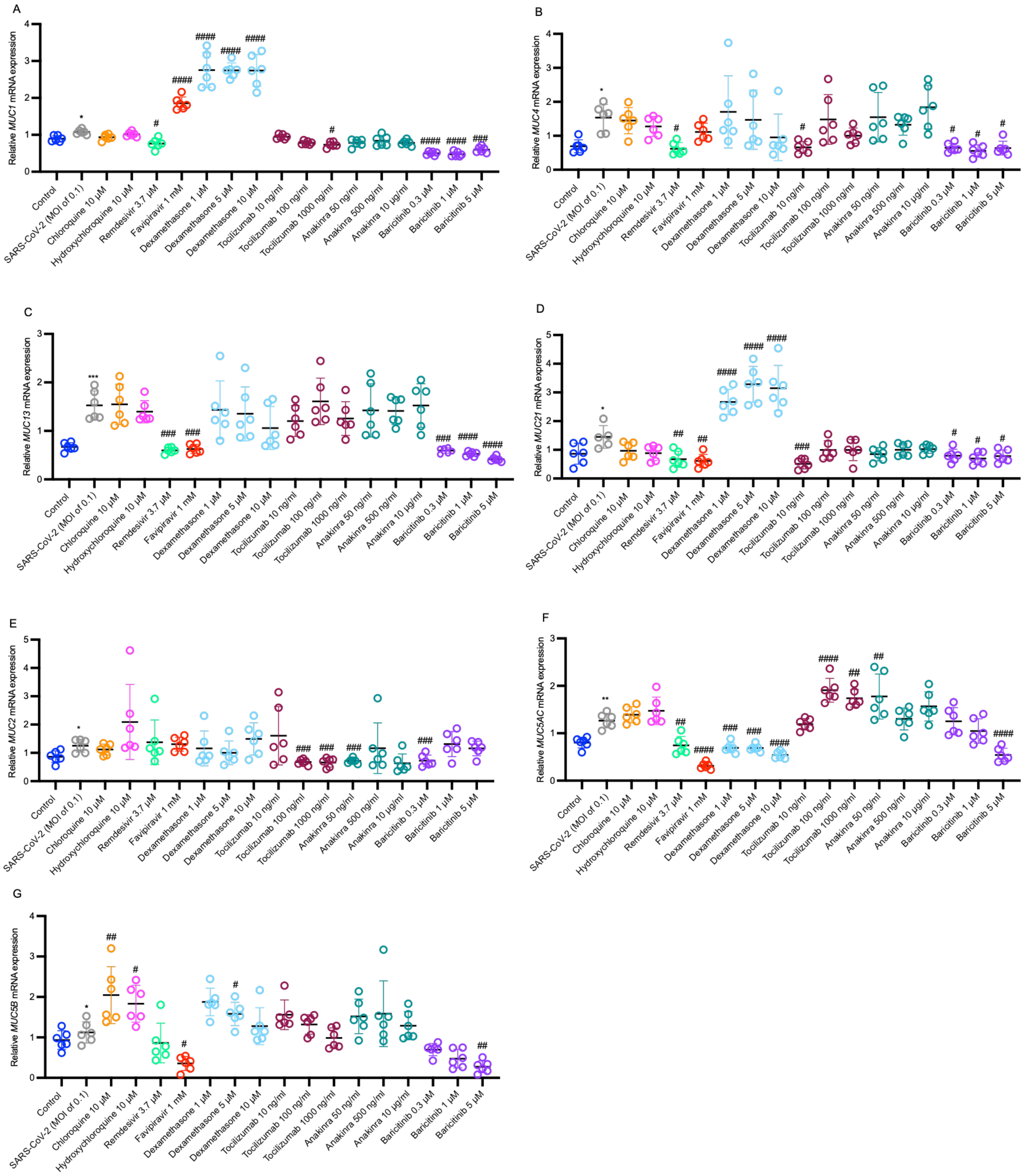

Supplementary Figure 4. relative mRNA expression data of *MUC1* (A), *MUC4* (B), MUC13 (C), MUC21 (D), MUC2 (E), *MUC5AC (F)* and *MUC5B (G)* in pulmonary (Calu3) epithelial cells infected with SARS-CoV-2 at 0.1 MOI for 2h and thereafter treated with a COVID-19 drug at different concentrations for 48h (n = 6). These include: remdesivir (3.7 μM); favipiravir (1mM), (hydroxy)chloroquine (10μM); dexamethasone (1, 5 and 10 μM); tocilizumab (10, 100 and 1000 ng/ml); anakinra (50 and 500 ng/ml, 10 μg/ml) and baricitinib (0.3, 1, 5 μM). SARS-CoV-2 infected untreated cells and uninfected/untreated cells were included as controls. Data are presented as mean ± SEM. Significant differences between treated and untreated cells upon SARS-CoV-2 infection are indicated by #p<0.05;

##$p<0.01$; ###$p<0{\cdot}001$; ####$p<0.0001$. Significant differences between SARS-CoV-2-infected and uninfected cells are indicated by *$p<0.05$; **$p<0.01$, ***$p<0.001$. One-Way ANOVA, Tukey’s post-hoc multiple comparison test.

Supplementary Table 1. Identification of variables associated with COVID-19 severity (i.e. critically ill or mild COVID-19) using Lasso regression

Variables	Coefficient	Variable importance[A]
**intercept**	-2.66	
**age**	0.064	85
**$\log_2$(MUC16)**	-0.53	85
**$\log_2$(MUC20)**	-0.28	85
**$\log_2$(MUC21)**	-0.008	30

[A] number of times a variable was selected in the optimal Lasso model for COVID-19 severity

Supplementary Table 2. Identification of variables associated with COVID-19 presentation using Lasso regression

Variables	Coefficient	Variable importance[A]
**intercept**	-0.55	
**age**	0.018	113
**$\log_2$(MUC1)**	0.35	112
**$\log_2$(MUC2)**	1.55	115
**$\log_2$(MUC4)**	-0.29	113
**$\log_2$(MUC6)**	-0.34	113
**$\log_2$(MUC13)**	0.12	39
**$\log_2$(MUC16)**	-0.73	115
**$\log_2$(MUC20)**	-0.10	114

[A] number of times a variable was selected in the optimal Lasso model for COVID-19 presentation

Supplementary Table 3. Prediction accuracy of multinomial regression analysis of the training set (n=80)

		**True outcome**			
		Critical covid-19	Mild covid-19	Mild non-covid-19	
**Predicted outcome**	Critical covid-19	34	4	1	**39**
	Mild covid-19	2	19	2	**23**
	Mild non-covid-19	0	3	15	**18**
		**36**	**26**	**18**	**80**

Supplementary Table 4. Prediction accuracy of multinomial regression analysis of the test set (n=35)

		**True outcome**			
		Critical covid-19	Mild covid-19	Mild non-covid-19	
**Predicted outcome**	Critical covid-19	10	3	1	**14**
	Mild covid-19	1	8	2	**11**
	Mild non-covid-19	0	0	10	**10**
		**11**	**11**	**13**	**35**
