# Supplementary material for: A dynamic mucin mRNA signature associates with COVID-19 disease presentation and severity
Source: JCI Insight. 2021 Oct 8;6(19):e151777. doi: 10.1172/jci.insight.151777 (PMC8525642; doi:10.1172/jci.insight.151777)
Supplement: ICMJE disclosure forms [file jciinsight-6-151777-s081.pdf]

ICMJE DISCLOSURE FORM

Date: 07 August 2021
Your Name: Annemieke Smet (corresponding author)
Manuscript Title: A dynamic mucin mRNA signature associates with COVID-19 disease presentation and severity
Manuscript number (if known): 151777-INS-CMED-TR-2

In the interest of transparency, we ask you to disclose all relationships/activities/interests listed below that are related to the content of your manuscript. "Related" means any relation with for-profit or not-for-profit third parties whose interests may be affected by the content of the manuscript. Disclosure represents a commitment to transparency and does not necessarily indicate a bias. If you are in doubt about whether to list a relationship/activity/interest, it is preferable that you do so.

The following questions apply to the author's relationships/activities/interests as they relate to the current manuscript only.

The author's relationships/activities/interests should be defined broadly. For example, if your manuscript pertains to the epidemiology of hypertension, you should declare all relationships with manufacturers of antihypertensive medication, even if that medication is not mentioned in the manuscript.

In item #1 below, report all support for the work reported in this manuscript without time limit. For all other items, the time frame for disclosure is the past 36 months.

		**Name all entities with whom you have this relationship or indicate none (add rows as needed)**	**Specifications/Comments (e.g., if payments were made to you or to your institution)**
		**Time frame: Since the initial planning of the work**	
1	All support for the present manuscript (e.g., funding, provision of study materials, medical writing, article processing charges, etc.) **No time limit for this item.**	the Antwerp University Research COVID-19 fund (BOF COVID-19; n° 42830)	
		the Research Foundation Flanders (FWO) COVID-19 funds (n° G0H3220N).	
		**Time frame: past 36 months**	
2	Grants or contracts from any entity (if not indicated in item #1 above).	see 1	
3	Royalties or licenses	patent (WO/2021/013479)	

4	Consulting fees	___X_ None	
5	Payment or honoraria for lectures, presentations, speakers bureaus, manuscript writing or educational events	__X__ None	
6	Payment for expert testimony	___X_ None	
7	Support for attending meetings and/or travel	___X_ None	
8	Patents planned, issued or pending	patent (WO/2021/013479)	
9	Participation on a Data Safety Monitoring Board or Advisory Board	__X__ None	
10	Leadership or fiduciary role in other board, society, committee or advocacy group, paid or unpaid	__X__ None	
11	Stock or stock options	___X_ None	
12	Receipt of equipment, materials, drugs, medical writing, gifts or other services	__X__ None	
13	Other financial or non-financial interests	__X__ None	

Please place an "X" next to the following statement to indicate your agreement:

__X_ I certify that I have answered every question and have not altered the wording of any of the questions on this form.

ICMJE DISCLOSURE FORM

Date: 14 August 2021
Your Name: Tom Breugelmans
Manuscript Title: A dynamic mucin mRNA signature associates with COVID-19 disease presentation and severity
Manuscript number (if known): 151777-INS-CMED-TR-2

In the interest of transparency, we ask you to disclose all relationships/activities/interests listed below that are related to the content of your manuscript. "Related" means any relation with for-profit or not-for-profit third parties whose interests may be affected by the content of the manuscript. Disclosure represents a commitment to transparency and does not necessarily indicate a bias. If you are in doubt about whether to list a relationship/activity/interest, it is preferable that you do so.

The following questions apply to the author's relationships/activities/interests as they relate to the current manuscript only.

The author's relationships/activities/interests should be defined broadly. For example, if your manuscript pertains to the epidemiology of hypertension, you should declare all relationships with manufacturers of antihypertensive medication, even if that medication is not mentioned in the manuscript.

In item #1 below, report all support for the work reported in this manuscript without time limit. For all other items, the time frame for disclosure is the past 36 months.

		**Name all entities with whom you have this relationship or indicate none (add rows as needed)**	**Specifications/Comments (e.g., if payments were made to you or to your institution)**
		**Time frame: Since the initial planning of the work**	
1	All support for the present manuscript (e.g., funding, provision of study materials, medical writing, article processing charges, etc.) **No time limit for this item.**	___X_ None	
		**Time frame: past 36 months**	
2	Grants or contracts from any entity (if not indicated in item #1 above).	___X_ None	
3	Royalties or licenses	patent (WO/2021/013479)	
4	Consulting fees	___X_ None	

5	Payment or honoraria for lectures, presentations, speakers bureaus, manuscript writing or educational events	__X__ None	
6	Payment for expert testimony	___X_ None	
7	Support for attending meetings and/or travel	___X_ None	
8	Patents planned, issued or pending	patent (WO/2021/013479)	
9	Participation on a Data Safety Monitoring Board or Advisory Board	__X__ None	
10	Leadership or fiduciary role in other board, society, committee or advocacy group, paid or unpaid	__X__ None	
11	Stock or stock options	___X_ None	
12	Receipt of equipment, materials, drugs, medical writing, gifts or other services	__X__ None	
13	Other financial or non-financial interests	__X__ None	

Please place an "X" next to the following statement to indicate your agreement:

__X_ I certify that I have answered every question and have not altered the wording of any of the questions on this form.

ICMJE DISCLOSURE FORM

Date: 14 August 2021
Your Name: Johan Michiels
Manuscript Title: A dynamic mucin mRNA signature associates with COVID-19 disease presentation and severity
Manuscript number (if known): 151777-INS-CMED-TR-2

In the interest of transparency, we ask you to disclose all relationships/activities/interests listed below that are related to the content of your manuscript. "Related" means any relation with for-profit or not-for-profit third parties whose interests may be affected by the content of the manuscript. Disclosure represents a commitment to transparency and does not necessarily indicate a bias. If you are in doubt about whether to list a relationship/activity/interest, it is preferable that you do so.

The following questions apply to the author's relationships/activities/interests as they relate to the current manuscript only.

The author's relationships/activities/interests should be defined broadly. For example, if your manuscript pertains to the epidemiology of hypertension, you should declare all relationships with manufacturers of antihypertensive medication, even if that medication is not mentioned in the manuscript.

In item #1 below, report all support for the work reported in this manuscript without time limit. For all other items, the time frame for disclosure is the past 36 months.

		**Name all entities with whom you have this relationship or indicate none (add rows as needed)**	**Specifications/Comments (e.g., if payments were made to you or to your institution)**
		**Time frame: Since the initial planning of the work**	
1	All support for the present manuscript (e.g., funding, provision of study materials, medical writing, article processing charges, etc.) **No time limit for this item.**	___X_ None	
		**Time frame: past 36 months**	
2	Grants or contracts from any entity (if not indicated in item #1 above).	___X_ None	
3	Royalties or licenses	___X_ None	

4	Consulting fees	___X_ None	
5	Payment or honoraria for lectures, presentations, speakers bureaus, manuscript writing or educational events	__X__ None	
6	Payment for expert testimony	___X_ None	
7	Support for attending meetings and/or travel	___X_ None	
8	Patents planned, issued or pending	___X_ None	
9	Participation on a Data Safety Monitoring Board or Advisory Board	__X__ None	
10	Leadership or fiduciary role in other board, society, committee or advocacy group, paid or unpaid	__X__ None	
11	Stock or stock options	___X_ None	
12	Receipt of equipment, materials, drugs, medical writing, gifts or other services	__X__ None	
13	Other financial or non-financial interests	__X__ None	

Please place an "X" next to the following statement to indicate your agreement:

__X_ I certify that I have answered every question and have not altered the wording of any of the questions on this form.

ICMJE DISCLOSURE FORM

Date: 14 August 2021
Your Name: Kevin Lamote
Manuscript Title: A dynamic mucin mRNA signature associates with COVID-19 disease presentation and severity
Manuscript number (if known): 151777-INS-CMED-TR-2

In the interest of transparency, we ask you to disclose all relationships/activities/interests listed below that are related to the content of your manuscript. "Related" means any relation with for-profit or not-for-profit third parties whose interests may be affected by the content of the manuscript. Disclosure represents a commitment to transparency and does not necessarily indicate a bias. If you are in doubt about whether to list a relationship/activity/interest, it is preferable that you do so.

The following questions apply to the author's relationships/activities/interests as they relate to the current manuscript only.

The author's relationships/activities/interests should be defined broadly. For example, if your manuscript pertains to the epidemiology of hypertension, you should declare all relationships with manufacturers of antihypertensive medication, even if that medication is not mentioned in the manuscript.

In item #1 below, report all support for the work reported in this manuscript without time limit. For all other items, the time frame for disclosure is the past 36 months.

		**Name all entities with whom you have this relationship or indicate none (add rows as needed)**	**Specifications/Comments (e.g., if payments were made to you or to your institution)**
		**Time frame: Since the initial planning of the work**	
1	All support for the present manuscript (e.g., funding, provision of study materials, medical writing, article processing charges, etc.) **No time limit for this item.**	___X_ None	
		**Time frame: past 36 months**	
2	Grants or contracts from any entity (if not indicated in item #1 above).	___X_ None	
3	Royalties or licenses	___X_ None	

4	Consulting fees	___X_ None	
5	Payment or honoraria for lectures, presentations, speakers bureaus, manuscript writing or educational events	__X__ None	
6	Payment for expert testimony	___X_ None	
7	Support for attending meetings and/or travel	___X_ None	
8	Patents planned, issued or pending	___X_ None	
9	Participation on a Data Safety Monitoring Board or Advisory Board	__X__ None	
10	Leadership or fiduciary role in other board, society, committee or advocacy group, paid or unpaid	__X__ None	
11	Stock or stock options	___X_ None	
12	Receipt of equipment, materials, drugs, medical writing, gifts or other services	__X__ None	
13	Other financial or non-financial interests	__X__ None	

Please place an "X" next to the following statement to indicate your agreement:

__X_ I certify that I have answered every question and have not altered the wording of any of the questions on this form.

ICMJE DISCLOSURE FORM

Date: 07 August 2021
Your Name: Wout Arras
Manuscript Title: A dynamic mucin mRNA signature associates with COVID-19 disease presentation and severity
Manuscript number (if known): 151777-INS-CMED-TR-2

In the interest of transparency, we ask you to disclose all relationships/activities/interests listed below that are related to the content of your manuscript. "Related" means any relation with for-profit or not-for-profit third parties whose interests may be affected by the content of the manuscript. Disclosure represents a commitment to transparency and does not necessarily indicate a bias. If you are in doubt about whether to list a relationship/activity/interest, it is preferable that you do so.

The following questions apply to the author's relationships/activities/interests as they relate to the current manuscript only.

The author's relationships/activities/interests should be defined broadly. For example, if your manuscript pertains to the epidemiology of hypertension, you should declare all relationships with manufacturers of antihypertensive medication, even if that medication is not mentioned in the manuscript.

In item #1 below, report all support for the work reported in this manuscript without time limit. For all other items, the time frame for disclosure is the past 36 months.

		**Name all entities with whom you have this relationship or indicate none (add rows as needed)**	**Specifications/Comments (e.g., if payments were made to you or to your institution)**
**Time frame: Since the initial planning of the work**			
1	All support for the present manuscript (e.g., funding, provision of study materials, medical writing, article processing charges, etc.) **No time limit for this item.**	___X_ None	
**Time frame: past 36 months**			
2	Grants or contracts from any entity (if not indicated in item #1 above).	___X_ None	
3	Royalties or licenses	___X_ None	

4	Consulting fees	___X_ None	
5	Payment or honoraria for lectures, presentations, speakers bureaus, manuscript writing or educational events	__X__ None	
6	Payment for expert testimony	___X_ None	
7	Support for attending meetings and/or travel	___X_ None	
8	Patents planned, issued or pending	___X_ None	
9	Participation on a Data Safety Monitoring Board or Advisory Board	__X__ None	
10	Leadership or fiduciary role in other board, society, committee or advocacy group, paid or unpaid	__X__ None	
11	Stock or stock options	___X_ None	
12	Receipt of equipment, materials, drugs, medical writing, gifts or other services	__X__ None	
13	Other financial or non-financial interests	__X__ None	

Please place an "X" next to the following statement to indicate your agreement:

__X_ I certify that I have answered every question and have not altered the wording of any of the questions on this form.

ICMJE DISCLOSURE FORM

Date: 14 August 2021
Your Name: Joris G. De Man
Manuscript Title: A dynamic mucin mRNA signature associates with COVID-19 disease presentation and severity
Manuscript number (if known): 151777-INS-CMED-TR-2

In the interest of transparency, we ask you to disclose all relationships/activities/interests listed below that are related to the content of your manuscript. "Related" means any relation with for-profit or not-for-profit third parties whose interests may be affected by the content of the manuscript. Disclosure represents a commitment to transparency and does not necessarily indicate a bias. If you are in doubt about whether to list a relationship/activity/interest, it is preferable that you do so.

The following questions apply to the author's relationships/activities/interests as they relate to the current manuscript only.

The author's relationships/activities/interests should be defined broadly. For example, if your manuscript pertains to the epidemiology of hypertension, you should declare all relationships with manufacturers of antihypertensive medication, even if that medication is not mentioned in the manuscript.

In item #1 below, report all support for the work reported in this manuscript without time limit. For all other items, the time frame for disclosure is the past 36 months.

		**Name all entities with whom you have this relationship or indicate none (add rows as needed)**	**Specifications/Comments (e.g., if payments were made to you or to your institution)**
		**Time frame: Since the initial planning of the work**	
1	All support for the present manuscript (e.g., funding, provision of study materials, medical writing, article processing charges, etc.) **No time limit for this item.**	___X_ None	
		**Time frame: past 36 months**	
2	Grants or contracts from any entity (if not indicated in item #1 above).	___X_ None	
3	Royalties or licenses	___X_ None	

4	Consulting fees	___X_ None	
5	Payment or honoraria for lectures, presentations, speakers bureaus, manuscript writing or educational events	__X__ None	
6	Payment for expert testimony	___X_ None	
7	Support for attending meetings and/or travel	___X_ None	
8	Patents planned, issued or pending	___X_ None	
9	Participation on a Data Safety Monitoring Board or Advisory Board	__X__ None	
10	Leadership or fiduciary role in other board, society, committee or advocacy group, paid or unpaid	__X__ None	
11	Stock or stock options	___X_ None	
12	Receipt of equipment, materials, drugs, medical writing, gifts or other services	__X__ None	
13	Other financial or non-financial interests	__X__ None	

Please place an "X" next to the following statement to indicate your agreement:

__X_ I certify that I have answered every question and have not altered the wording of any of the questions on this form.

ICMJE DISCLOSURE FORM

Date: 14 August 2021
Your Name: Leo Heyndrickx
Manuscript Title: A dynamic mucin mRNA signature associates with COVID-19 disease presentation and severity
Manuscript number (if known): 151777-INS-CMED-TR-2

In the interest of transparency, we ask you to disclose all relationships/activities/interests listed below that are related to the content of your manuscript. "Related" means any relation with for-profit or not-for-profit third parties whose interests may be affected by the content of the manuscript. Disclosure represents a commitment to transparency and does not necessarily indicate a bias. If you are in doubt about whether to list a relationship/activity/interest, it is preferable that you do so.

The following questions apply to the author's relationships/activities/interests as they relate to the current manuscript only.

The author's relationships/activities/interests should be defined broadly. For example, if your manuscript pertains to the epidemiology of hypertension, you should declare all relationships with manufacturers of antihypertensive medication, even if that medication is not mentioned in the manuscript.

In item #1 below, report all support for the work reported in this manuscript without time limit. For all other items, the time frame for disclosure is the past 36 months.

		**Name all entities with whom you have this relationship or indicate none (add rows as needed)**	**Specifications/Comments (e.g., if payments were made to you or to your institution)**
**Time frame: Since the initial planning of the work**			
1	All support for the present manuscript (e.g., funding, provision of study materials, medical writing, article processing charges, etc.) **No time limit for this item.**	___X_ None	
**Time frame: past 36 months**			
2	Grants or contracts from any entity (if not indicated in item #1 above).	___X_ None	
3	Royalties or licenses	___X_ None	

4	Consulting fees	___X_ None	
5	Payment or honoraria for lectures, presentations, speakers bureaus, manuscript writing or educational events	__X__ None	
6	Payment for expert testimony	___X_ None	
7	Support for attending meetings and/or travel	___X_ None	
8	Patents planned, issued or pending	___X_ None	
9	Participation on a Data Safety Monitoring Board or Advisory Board	__X__ None	
10	Leadership or fiduciary role in other board, society, committee or advocacy group, paid or unpaid	__X__ None	
11	Stock or stock options	___X_ None	
12	Receipt of equipment, materials, drugs, medical writing, gifts or other services	__X__ None	
13	Other financial or non-financial interests	__X__ None	

Please place an "X" next to the following statement to indicate your agreement:

__X_ I certify that I have answered every question and have not altered the wording of any of the questions on this form.

ICMJE DISCLOSURE FORM

Date: 14 August 2021
Your Name: Anne Hauner
Manuscript Title: A dynamic mucin mRNA signature associates with COVID-19 disease presentation and severity
Manuscript number (if known): 151777-INS-CMED-TR-2

In the interest of transparency, we ask you to disclose all relationships/activities/interests listed below that are related to the content of your manuscript. "Related" means any relation with for-profit or not-for-profit third parties whose interests may be affected by the content of the manuscript. Disclosure represents a commitment to transparency and does not necessarily indicate a bias. If you are in doubt about whether to list a relationship/activity/interest, it is preferable that you do so.

The following questions apply to the author's relationships/activities/interests as they relate to the current manuscript only.

The author's relationships/activities/interests should be defined broadly. For example, if your manuscript pertains to the epidemiology of hypertension, you should declare all relationships with manufacturers of antihypertensive medication, even if that medication is not mentioned in the manuscript.

In item #1 below, report all support for the work reported in this manuscript without time limit. For all other items, the time frame for disclosure is the past 36 months.

		**Name all entities with whom you have this relationship or indicate none (add rows as needed)**	**Specifications/Comments (e.g., if payments were made to you or to your institution)**
		**Time frame: Since the initial planning of the work**	
1	All support for the present manuscript (e.g., funding, provision of study materials, medical writing, article processing charges, etc.) **No time limit for this item.**	___X_ None	
		**Time frame: past 36 months**	
2	Grants or contracts from any entity (if not indicated in item #1 above).	___X_ None	
3	Royalties or licenses	___X_ None	

4	Consulting fees	___X_ None	
5	Payment or honoraria for lectures, presentations, speakers bureaus, manuscript writing or educational events	__X__ None	
6	Payment for expert testimony	___X_ None	
7	Support for attending meetings and/or travel	___X_ None	
8	Patents planned, issued or pending	___X_ None	
9	Participation on a Data Safety Monitoring Board or Advisory Board	__X__ None	
10	Leadership or fiduciary role in other board, society, committee or advocacy group, paid or unpaid	__X__ None	
11	Stock or stock options	___X_ None	
12	Receipt of equipment, materials, drugs, medical writing, gifts or other services	__X__ None	
13	Other financial or non-financial interests	__X__ None	

Please place an "X" next to the following statement to indicate your agreement:

__X_ I certify that I have answered every question and have not altered the wording of any of the questions on this form.

ICMJE DISCLOSURE FORM

Date: 14 August 2021
Your Name: Manon Huizing
Manuscript Title: A dynamic mucin mRNA signature associates with COVID-19 disease presentation and severity
Manuscript number (if known): 151777-INS-CMED-TR-2

In the interest of transparency, we ask you to disclose all relationships/activities/interests listed below that are related to the content of your manuscript. "Related" means any relation with for-profit or not-for-profit third parties whose interests may be affected by the content of the manuscript. Disclosure represents a commitment to transparency and does not necessarily indicate a bias. If you are in doubt about whether to list a relationship/activity/interest, it is preferable that you do so.

The following questions apply to the author's relationships/activities/interests as they relate to the current manuscript only.

The author's relationships/activities/interests should be defined broadly. For example, if your manuscript pertains to the epidemiology of hypertension, you should declare all relationships with manufacturers of antihypertensive medication, even if that medication is not mentioned in the manuscript.

In item #1 below, report all support for the work reported in this manuscript without time limit. For all other items, the time frame for disclosure is the past 36 months.

		**Name all entities with whom you have this relationship or indicate none (add rows as needed)**	**Specifications/Comments (e.g., if payments were made to you or to your institution)**
**Time frame: Since the initial planning of the work**			
1	All support for the present manuscript (e.g., funding, provision of study materials, medical writing, article processing charges, etc.) **No time limit for this item.**	___X_ None	
**Time frame: past 36 months**			
2	Grants or contracts from any entity (if not indicated in item #1 above).	___X_ None	
3	Royalties or licenses	___X_ None	

4	Consulting fees	___X_ None	
5	Payment or honoraria for lectures, presentations, speakers bureaus, manuscript writing or educational events	__X__ None	
6	Payment for expert testimony	___X_ None	
7	Support for attending meetings and/or travel	___X_ None	
8	Patents planned, issued or pending	___X_ None	
9	Participation on a Data Safety Monitoring Board or Advisory Board	__X__ None	
10	Leadership or fiduciary role in other board, society, committee or advocacy group, paid or unpaid	__X__ None	
11	Stock or stock options	___X_ None	
12	Receipt of equipment, materials, drugs, medical writing, gifts or other services	__X__ None	
13	Other financial or non-financial interests	__X__ None	

Please place an "X" next to the following statement to indicate your agreement:

__X_ I certify that I have answered every question and have not altered the wording of any of the questions on this form.

ICMJE DISCLOSURE FORM

Date: 14 August 2021
Your Name: Surbhi Malhotra-Kumar
Manuscript Title: A dynamic mucin mRNA signature associates with COVID-19 disease presentation and severity
Manuscript number (if known): 151777-INS-CMED-TR-2

In the interest of transparency, we ask you to disclose all relationships/activities/interests listed below that are related to the content of your manuscript. "Related" means any relation with for-profit or not-for-profit third parties whose interests may be affected by the content of the manuscript. Disclosure represents a commitment to transparency and does not necessarily indicate a bias. If you are in doubt about whether to list a relationship/activity/interest, it is preferable that you do so.

The following questions apply to the author's relationships/activities/interests as they relate to the current manuscript only.

The author's relationships/activities/interests should be defined broadly. For example, if your manuscript pertains to the epidemiology of hypertension, you should declare all relationships with manufacturers of antihypertensive medication, even if that medication is not mentioned in the manuscript.

In item #1 below, report all support for the work reported in this manuscript without time limit. For all other items, the time frame for disclosure is the past 36 months.

		**Name all entities with whom you have this relationship or indicate none (add rows as needed)**	**Specifications/Comments (e.g., if payments were made to you or to your institution)**
		**Time frame: Since the initial planning of the work**	
1	All support for the present manuscript (e.g., funding, provision of study materials, medical writing, article processing charges, etc.) **No time limit for this item.**	the Antwerp University Research COVID-19 fund (BOF COVID-19; n° 42830)	
		the Research Foundation Flanders (FWO) COVID-19 funds (n° G0H3220N).	
		**Time frame: past 36 months**	
2	Grants or contracts from any entity (if not indicated in item #1 above).	see 1	
3	Royalties or licenses	___X_ None	

4	Consulting fees	___X_ None	
5	Payment or honoraria for lectures, presentations, speakers bureaus, manuscript writing or educational events	__X__ None	
6	Payment for expert testimony	___X_ None	
7	Support for attending meetings and/or travel	___X_ None	
8	Patents planned, issued or pending	___X_ None	
9	Participation on a Data Safety Monitoring Board or Advisory Board	__X__ None	
10	Leadership or fiduciary role in other board, society, committee or advocacy group, paid or unpaid	__X__ None	
11	Stock or stock options	___X_ None	
12	Receipt of equipment, materials, drugs, medical writing, gifts or other services	__X__ None	
13	Other financial or non-financial interests	__X__ None	

Please place an "X" next to the following statement to indicate your agreement:

__X_ I certify that I have answered every question and have not altered the wording of any of the questions on this form.

ICMJE DISCLOSURE FORM

Date: 14 August 2021
Your Name: Martin Lammens
Manuscript Title: A dynamic mucin mRNA signature associates with COVID-19 disease presentation and severity
Manuscript number (if known): 151777-INS-CMED-TR-2

In the interest of transparency, we ask you to disclose all relationships/activities/interests listed below that are related to the content of your manuscript. "Related" means any relation with for-profit or not-for-profit third parties whose interests may be affected by the content of the manuscript. Disclosure represents a commitment to transparency and does not necessarily indicate a bias. If you are in doubt about whether to list a relationship/activity/interest, it is preferable that you do so.

The following questions apply to the author's relationships/activities/interests as they relate to the current manuscript only.

The author's relationships/activities/interests should be defined broadly. For example, if your manuscript pertains to the epidemiology of hypertension, you should declare all relationships with manufacturers of antihypertensive medication, even if that medication is not mentioned in the manuscript.

In item #1 below, report all support for the work reported in this manuscript without time limit. For all other items, the time frame for disclosure is the past 36 months.

		**Name all entities with whom you have this relationship or indicate none (add rows as needed)**	**Specifications/Comments (e.g., if payments were made to you or to your institution)**
		**Time frame: Since the initial planning of the work**	
1	All support for the present manuscript (e.g., funding, provision of study materials, medical writing, article processing charges, etc.) **No time limit for this item.**	___X_ None	
		**Time frame: past 36 months**	
2	Grants or contracts from any entity (if not indicated in item #1 above).	___X_ None	
3	Royalties or licenses	___X_ None	

4	Consulting fees	___X_ None	
5	Payment or honoraria for lectures, presentations, speakers bureaus, manuscript writing or educational events	__X__ None	
6	Payment for expert testimony	___X_ None	
7	Support for attending meetings and/or travel	___X_ None	
8	Patents planned, issued or pending	___X_ None	
9	Participation on a Data Safety Monitoring Board or Advisory Board	__X__ None	
10	Leadership or fiduciary role in other board, society, committee or advocacy group, paid or unpaid	__X__ None	
11	Stock or stock options	___X_ None	
12	Receipt of equipment, materials, drugs, medical writing, gifts or other services	__X__ None	
13	Other financial or non-financial interests	__X__ None	

Please place an "X" next to the following statement to indicate your agreement:

__X_ I certify that I have answered every question and have not altered the wording of any of the questions on this form.

ICMJE DISCLOSURE FORM

Date: 14 August 2021
Your Name: An Hotterbeekx
Manuscript Title: A dynamic mucin mRNA signature associates with COVID-19 disease presentation and severity
Manuscript number (if known): 151777-INS-CMED-TR-2

In the interest of transparency, we ask you to disclose all relationships/activities/interests listed below that are related to the content of your manuscript. "Related" means any relation with for-profit or not-for-profit third parties whose interests may be affected by the content of the manuscript. Disclosure represents a commitment to transparency and does not necessarily indicate a bias. If you are in doubt about whether to list a relationship/activity/interest, it is preferable that you do so.

The following questions apply to the author's relationships/activities/interests as they relate to the <u>current manuscript only</u>.

The author's relationships/activities/interests should be <u>defined broadly</u>. For example, if your manuscript pertains to the epidemiology of hypertension, you should declare all relationships with manufacturers of antihypertensive medication, even if that medication is not mentioned in the manuscript.

In item #1 below, report all support for the work reported in this manuscript without time limit. For all other items, the time frame for disclosure is the past 36 months.

		Name all entities with whom you have this relationship or indicate none (add rows as needed)	Specifications/Comments (e.g., if payments were made to you or to your institution)
**Time frame: Since the initial planning of the work**			
1	All support for the present manuscript (e.g., funding, provision of study materials, medical writing, article processing charges, etc.) **No time limit for this item.**	___X_ None	
**Time frame: past 36 months**			
2	Grants or contracts from any entity (if not indicated in item #1 above).	___X_ None	
3	Royalties or licenses	___X_ None	

4	Consulting fees	___X_ None	
5	Payment or honoraria for lectures, presentations, speakers bureaus, manuscript writing or educational events	__X__ None	
6	Payment for expert testimony	___X_ None	
7	Support for attending meetings and/or travel	___X_ None	
8	Patents planned, issued or pending	___X_ None	
9	Participation on a Data Safety Monitoring Board or Advisory Board	__X__ None	
10	Leadership or fiduciary role in other board, society, committee or advocacy group, paid or unpaid	__X__ None	
11	Stock or stock options	___X_ None	
12	Receipt of equipment, materials, drugs, medical writing, gifts or other services	__X__ None	
13	Other financial or non-financial interests	__X__ None	

Please place an "X" next to the following statement to indicate your agreement:

__X_ I certify that I have answered every question and have not altered the wording of any of the questions on this form.

ICMJE DISCLOSURE FORM

Date: 14 August 2021
Your Name: Samir Kumar-Singh
Manuscript Title: A dynamic mucin mRNA signature associates with COVID-19 disease presentation and severity
Manuscript number (if known): 151777-INS-CMED-TR-2

In the interest of transparency, we ask you to disclose all relationships/activities/interests listed below that are related to the content of your manuscript. "Related" means any relation with for-profit or not-for-profit third parties whose interests may be affected by the content of the manuscript. Disclosure represents a commitment to transparency and does not necessarily indicate a bias. If you are in doubt about whether to list a relationship/activity/interest, it is preferable that you do so.

The following questions apply to the author's relationships/activities/interests as they relate to the current manuscript only.

The author's relationships/activities/interests should be defined broadly. For example, if your manuscript pertains to the epidemiology of hypertension, you should declare all relationships with manufacturers of antihypertensive medication, even if that medication is not mentioned in the manuscript.

In item #1 below, report all support for the work reported in this manuscript without time limit. For all other items, the time frame for disclosure is the past 36 months.

		**Name all entities with whom you have this relationship or indicate none (add rows as needed)**	**Specifications/Comments (e.g., if payments were made to you or to your institution)**
		**Time frame: Since the initial planning of the work**	
1	All support for the present manuscript (e.g., funding, provision of study materials, medical writing, article processing charges, etc.) **No time limit for this item.**	the Antwerp University Research COVID-19 fund (BOF COVID-19; n° 42830)	
		**Time frame: past 36 months**	
2	Grants or contracts from any entity (if not indicated in item #1 above).	see 1	
3	Royalties or licenses	___X_ None	

4	Consulting fees	___X_ None	
5	Payment or honoraria for lectures, presentations, speakers bureaus, manuscript writing or educational events	__X__ None	
6	Payment for expert testimony	___X_ None	
7	Support for attending meetings and/or travel	___X_ None	
8	Patents planned, issued or pending	___X_ None	
9	Participation on a Data Safety Monitoring Board or Advisory Board	__X__ None	
10	Leadership or fiduciary role in other board, society, committee or advocacy group, paid or unpaid	__X__ None	
11	Stock or stock options	___X_ None	
12	Receipt of equipment, materials, drugs, medical writing, gifts or other services	__X__ None	
13	Other financial or non-financial interests	__X__ None	

Please place an "X" next to the following statement to indicate your agreement:

__X_ I certify that I have answered every question and have not altered the wording of any of the questions on this form.

ICMJE DISCLOSURE FORM

Date: 14 August 2021
Your Name: Aline Verstraeten
Manuscript Title: A dynamic mucin mRNA signature associates with COVID-19 disease presentation and severity
Manuscript number (if known): 151777-INS-CMED-TR-2

In the interest of transparency, we ask you to disclose all relationships/activities/interests listed below that are related to the content of your manuscript. "Related" means any relation with for-profit or not-for-profit third parties whose interests may be affected by the content of the manuscript. Disclosure represents a commitment to transparency and does not necessarily indicate a bias. If you are in doubt about whether to list a relationship/activity/interest, it is preferable that you do so.

The following questions apply to the author's relationships/activities/interests as they relate to the current manuscript only.

The author's relationships/activities/interests should be defined broadly. For example, if your manuscript pertains to the epidemiology of hypertension, you should declare all relationships with manufacturers of antihypertensive medication, even if that medication is not mentioned in the manuscript.

In item #1 below, report all support for the work reported in this manuscript without time limit. For all other items, the time frame for disclosure is the past 36 months.

		**Name all entities with whom you have this relationship or indicate none (add rows as needed)**	**Specifications/Comments (e.g., if payments were made to you or to your institution)**
		**Time frame: Since the initial planning of the work**	
1	All support for the present manuscript (e.g., funding, provision of study materials, medical writing, article processing charges, etc.) **No time limit for this item.**	the Antwerp University Research COVID-19 fund (BOF COVID-19; n° 42830)	
		the Research Foundation Flanders (FWO) COVID-19 funds (n° G0H3220N).	
		**Time frame: past 36 months**	
2	Grants or contracts from any entity (if not indicated in item #1 above).	see 1	
3	Royalties or licenses	___X_ None	

4	Consulting fees	___X_ None	
5	Payment or honoraria for lectures, presentations, speakers bureaus, manuscript writing or educational events	__X__ None	
6	Payment for expert testimony	___X_ None	
7	Support for attending meetings and/or travel	___X_ None	
8	Patents planned, issued or pending	___X_ None	
9	Participation on a Data Safety Monitoring Board or Advisory Board	__X__ None	
10	Leadership or fiduciary role in other board, society, committee or advocacy group, paid or unpaid	__X__ None	
11	Stock or stock options	___X_ None	
12	Receipt of equipment, materials, drugs, medical writing, gifts or other services	__X__ None	
13	Other financial or non-financial interests	__X__ None	

Please place an "X" next to the following statement to indicate your agreement:

__X_ I certify that I have answered every question and have not altered the wording of any of the questions on this form.

ICMJE DISCLOSURE FORM

Date: 14 August 2021
Your Name: Bart Loeys
Manuscript Title: A dynamic mucin mRNA signature associates with COVID-19 disease presentation and severity
Manuscript number (if known): 151777-INS-CMED-TR-2

In the interest of transparency, we ask you to disclose all relationships/activities/interests listed below that are related to the content of your manuscript. "Related" means any relation with for-profit or not-for-profit third parties whose interests may be affected by the content of the manuscript. Disclosure represents a commitment to transparency and does not necessarily indicate a bias. If you are in doubt about whether to list a relationship/activity/interest, it is preferable that you do so.

The following questions apply to the author's relationships/activities/interests as they relate to the current manuscript only.

The author's relationships/activities/interests should be defined broadly. For example, if your manuscript pertains to the epidemiology of hypertension, you should declare all relationships with manufacturers of antihypertensive medication, even if that medication is not mentioned in the manuscript.

In item #1 below, report all support for the work reported in this manuscript without time limit. For all other items, the time frame for disclosure is the past 36 months.

		**Name all entities with whom you have this relationship or indicate none (add rows as needed)**	**Specifications/Comments (e.g., if payments were made to you or to your institution)**
		**Time frame: Since the initial planning of the work**	
1	All support for the present manuscript (e.g., funding, provision of study materials, medical writing, article processing charges, etc.) **No time limit for this item.**	___X_ None	
		**Time frame: past 36 months**	
2	Grants or contracts from any entity (if not indicated in item #1 above).	___X_ None	
3	Royalties or licenses	___X_ None	

4	Consulting fees	___X_ None	
5	Payment or honoraria for lectures, presentations, speakers bureaus, manuscript writing or educational events	__X__ None	
6	Payment for expert testimony	___X_ None	
7	Support for attending meetings and/or travel	___X_ None	
8	Patents planned, issued or pending	___X_ None	
9	Participation on a Data Safety Monitoring Board or Advisory Board	__X__ None	
10	Leadership or fiduciary role in other board, society, committee or advocacy group, paid or unpaid	__X__ None	
11	Stock or stock options	___X_ None	
12	Receipt of equipment, materials, drugs, medical writing, gifts or other services	__X__ None	
13	Other financial or non-financial interests	__X__ None	

Please place an “X” next to the following statement to indicate your agreement:

__X_ I certify that I have answered every question and have not altered the wording of any of the questions on this form.

ICMJE DISCLOSURE FORM

Date: 14 August 2021
Your Name: Veronique Verhoeven
Manuscript Title: A dynamic mucin mRNA signature associates with COVID-19 disease presentation and severity
Manuscript number (if known): 151777-INS-CMED-TR-2

In the interest of transparency, we ask you to disclose all relationships/activities/interests listed below that are related to the content of your manuscript. "Related" means any relation with for-profit or not-for-profit third parties whose interests may be affected by the content of the manuscript. Disclosure represents a commitment to transparency and does not necessarily indicate a bias. If you are in doubt about whether to list a relationship/activity/interest, it is preferable that you do so.

The following questions apply to the author's relationships/activities/interests as they relate to the current manuscript only.

The author's relationships/activities/interests should be defined broadly. For example, if your manuscript pertains to the epidemiology of hypertension, you should declare all relationships with manufacturers of antihypertensive medication, even if that medication is not mentioned in the manuscript.

In item #1 below, report all support for the work reported in this manuscript without time limit. For all other items, the time frame for disclosure is the past 36 months.

		**Name all entities with whom you have this relationship or indicate none (add rows as needed)**	**Specifications/Comments (e.g., if payments were made to you or to your institution)**
**Time frame: Since the initial planning of the work**			
1	All support for the present manuscript (e.g., funding, provision of study materials, medical writing, article processing charges, etc.) **No time limit for this item.**	___X_ None	
**Time frame: past 36 months**			
2	Grants or contracts from any entity (if not indicated in item #1 above).	___X_ None	
3	Royalties or licenses	___X_ None	

4	Consulting fees	___X_ None	
5	Payment or honoraria for lectures, presentations, speakers bureaus, manuscript writing or educational events	__X__ None	
6	Payment for expert testimony	___X_ None	
7	Support for attending meetings and/or travel	___X_ None	
8	Patents planned, issued or pending	___X_ None	
9	Participation on a Data Safety Monitoring Board or Advisory Board	__X__ None	
10	Leadership or fiduciary role in other board, society, committee or advocacy group, paid or unpaid	__X__ None	
11	Stock or stock options	___X_ None	
12	Receipt of equipment, materials, drugs, medical writing, gifts or other services	__X__ None	
13	Other financial or non-financial interests	__X__ None	

Please place an "X" next to the following statement to indicate your agreement:

__X_ I certify that I have answered every question and have not altered the wording of any of the questions on this form.

ICMJE DISCLOSURE FORM

Date: 14 August 2021
Your Name: Rita Jacobs
Manuscript Title: A dynamic mucin mRNA signature associates with COVID-19 disease presentation and severity
Manuscript number (if known): 151777-INS-CMED-TR-2

In the interest of transparency, we ask you to disclose all relationships/activities/interests listed below that are related to the content of your manuscript. "Related" means any relation with for-profit or not-for-profit third parties whose interests may be affected by the content of the manuscript. Disclosure represents a commitment to transparency and does not necessarily indicate a bias. If you are in doubt about whether to list a relationship/activity/interest, it is preferable that you do so.

The following questions apply to the author's relationships/activities/interests as they relate to the current manuscript only.

The author's relationships/activities/interests should be defined broadly. For example, if your manuscript pertains to the epidemiology of hypertension, you should declare all relationships with manufacturers of antihypertensive medication, even if that medication is not mentioned in the manuscript.

In item #1 below, report all support for the work reported in this manuscript without time limit. For all other items, the time frame for disclosure is the past 36 months.

		**Name all entities with whom you have this relationship or indicate none (add rows as needed)**	**Specifications/Comments (e.g., if payments were made to you or to your institution)**
		**Time frame: Since the initial planning of the work**	
1	All support for the present manuscript (e.g., funding, provision of study materials, medical writing, article processing charges, etc.) **No time limit for this item.**	___X_ None	
		**Time frame: past 36 months**	
2	Grants or contracts from any entity (if not indicated in item #1 above).	___X_ None	
3	Royalties or licenses	___X_ None	

4	Consulting fees	___X_ None	
5	Payment or honoraria for lectures, presentations, speakers bureaus, manuscript writing or educational events	__X__ None	
6	Payment for expert testimony	___X_ None	
7	Support for attending meetings and/or travel	___X_ None	
8	Patents planned, issued or pending	___X_ None	
9	Participation on a Data Safety Monitoring Board or Advisory Board	__X__ None	
10	Leadership or fiduciary role in other board, society, committee or advocacy group, paid or unpaid	__X__ None	
11	Stock or stock options	___X_ None	
12	Receipt of equipment, materials, drugs, medical writing, gifts or other services	__X__ None	
13	Other financial or non-financial interests	__X__ None	

Please place an "X" next to the following statement to indicate your agreement:

__X_ I certify that I have answered every question and have not altered the wording of any of the questions on this form.

ICMJE DISCLOSURE FORM

Date: 14 August 2021
Your Name: Karolien Dams
Manuscript Title: A dynamic mucin mRNA signature associates with COVID-19 disease presentation and severity
Manuscript number (if known): 151777-INS-CMED-TR-2

In the interest of transparency, we ask you to disclose all relationships/activities/interests listed below that are related to the content of your manuscript. "Related" means any relation with for-profit or not-for-profit third parties whose interests may be affected by the content of the manuscript. Disclosure represents a commitment to transparency and does not necessarily indicate a bias. If you are in doubt about whether to list a relationship/activity/interest, it is preferable that you do so.

The following questions apply to the author's relationships/activities/interests as they relate to the current manuscript only.

The author's relationships/activities/interests should be defined broadly. For example, if your manuscript pertains to the epidemiology of hypertension, you should declare all relationships with manufacturers of antihypertensive medication, even if that medication is not mentioned in the manuscript.

In item #1 below, report all support for the work reported in this manuscript without time limit. For all other items, the time frame for disclosure is the past 36 months.

		**Name all entities with whom you have this relationship or indicate none (add rows as needed)**	**Specifications/Comments (e.g., if payments were made to you or to your institution)**
		**Time frame: Since the initial planning of the work**	
1	All support for the present manuscript (e.g., funding, provision of study materials, medical writing, article processing charges, etc.) **No time limit for this item.**	___X_ None	
		**Time frame: past 36 months**	
2	Grants or contracts from any entity (if not indicated in item #1 above).	___X_ None	
3	Royalties or licenses	___X_ None	

4	Consulting fees	___X_ None	
5	Payment or honoraria for lectures, presentations, speakers bureaus, manuscript writing or educational events	__X__ None	
6	Payment for expert testimony	___X_ None	
7	Support for attending meetings and/or travel	___X_ None	
8	Patents planned, issued or pending	___X_ None	
9	Participation on a Data Safety Monitoring Board or Advisory Board	__X__ None	
10	Leadership or fiduciary role in other board, society, committee or advocacy group, paid or unpaid	__X__ None	
11	Stock or stock options	___X_ None	
12	Receipt of equipment, materials, drugs, medical writing, gifts or other services	__X__ None	
13	Other financial or non-financial interests	__X__ None	

Please place an "X" next to the following statement to indicate your agreement:

__X_ I certify that I have answered every question and have not altered the wording of any of the questions on this form.

ICMJE DISCLOSURE FORM

Date: 14 August 2021
Your Name: Samuel Coenen
Manuscript Title: A dynamic mucin mRNA signature associates with COVID-19 disease presentation and severity
Manuscript number (if known): 151777-INS-CMED-TR-2

In the interest of transparency, we ask you to disclose all relationships/activities/interests listed below that are related to the content of your manuscript. "Related" means any relation with for-profit or not-for-profit third parties whose interests may be affected by the content of the manuscript. Disclosure represents a commitment to transparency and does not necessarily indicate a bias. If you are in doubt about whether to list a relationship/activity/interest, it is preferable that you do so.

The following questions apply to the author's relationships/activities/interests as they relate to the <u>current manuscript only</u>.

The author's relationships/activities/interests should be <u>defined broadly</u>. For example, if your manuscript pertains to the epidemiology of hypertension, you should declare all relationships with manufacturers of antihypertensive medication, even if that medication is not mentioned in the manuscript.

In item #1 below, report all support for the work reported in this manuscript without time limit. For all other items, the time frame for disclosure is the past 36 months.

		**Name all entities with whom you have this relationship or indicate none (add rows as needed)**	**Specifications/Comments (e.g., if payments were made to you or to your institution)**
		**Time frame: Since the initial planning of the work**	
1	All support for the present manuscript (e.g., funding, provision of study materials, medical writing, article processing charges, etc.) **No time limit for this item.**	___X_ None	
		**Time frame: past 36 months**	
2	Grants or contracts from any entity (if not indicated in item #1 above).	___X_ None	
3	Royalties or licenses	___X_ None	

4	Consulting fees	___X_ None	
5	Payment or honoraria for lectures, presentations, speakers bureaus, manuscript writing or educational events	__X__ None	
6	Payment for expert testimony	___X_ None	
7	Support for attending meetings and/or travel	___X_ None	
8	Patents planned, issued or pending	___X_ None	
9	Participation on a Data Safety Monitoring Board or Advisory Board	__X__ None	
10	Leadership or fiduciary role in other board, society, committee or advocacy group, paid or unpaid	__X__ None	
11	Stock or stock options	___X_ None	
12	Receipt of equipment, materials, drugs, medical writing, gifts or other services	__X__ None	
13	Other financial or non-financial interests	__X__ None	

Please place an "X" next to the following statement to indicate your agreement:

__X_ I certify that I have answered every question and have not altered the wording of any of the questions on this form.

ICMJE DISCLOSURE FORM

Date: 14 August 2021
Your Name: Kevin K. Ariën
Manuscript Title: A dynamic mucin mRNA signature associates with COVID-19 disease presentation and severity
Manuscript number (if known): 151777-INS-CMED-TR-2

In the interest of transparency, we ask you to disclose all relationships/activities/interests listed below that are related to the content of your manuscript. "Related" means any relation with for-profit or not-for-profit third parties whose interests may be affected by the content of the manuscript. Disclosure represents a commitment to transparency and does not necessarily indicate a bias. If you are in doubt about whether to list a relationship/activity/interest, it is preferable that you do so.

The following questions apply to the author's relationships/activities/interests as they relate to the current manuscript only.

The author's relationships/activities/interests should be defined broadly. For example, if your manuscript pertains to the epidemiology of hypertension, you should declare all relationships with manufacturers of antihypertensive medication, even if that medication is not mentioned in the manuscript.

In item #1 below, report all support for the work reported in this manuscript without time limit. For all other items, the time frame for disclosure is the past 36 months.

		**Name all entities with whom you have this relationship or indicate none (add rows as needed)**	**Specifications/Comments (e.g., if payments were made to you or to your institution)**
**Time frame: Since the initial planning of the work**			
1	All support for the present manuscript (e.g., funding, provision of study materials, medical writing, article processing charges, etc.) **No time limit for this item.**	the Research Foundation Flanders (FWO) COVID-19 funds (n° G0H3220N; and n° G0G4220N).	
**Time frame: past 36 months**			
2	Grants or contracts from any entity (if not indicated in item #1 above).	see 1	
3	Royalties or licenses	___X_ None	

4	Consulting fees	___X_ None	
5	Payment or honoraria for lectures, presentations, speakers bureaus, manuscript writing or educational events	__X__ None	
6	Payment for expert testimony	___X_ None	
7	Support for attending meetings and/or travel	___X_ None	
8	Patents planned, issued or pending	___X_ None	
9	Participation on a Data Safety Monitoring Board or Advisory Board	__X__ None	
10	Leadership or fiduciary role in other board, society, committee or advocacy group, paid or unpaid	__X__ None	
11	Stock or stock options	___X_ None	
12	Receipt of equipment, materials, drugs, medical writing, gifts or other services	__X__ None	
13	Other financial or non-financial interests	__X__ None	

Please place an "X" next to the following statement to indicate your agreement:

__X_ I certify that I have answered every question and have not altered the wording of any of the questions on this form.

ICMJE DISCLOSURE FORM

Date: 14 August 2021
Your Name: Philippe G. Jorens
Manuscript Title: A dynamic mucin mRNA signature associates with COVID-19 disease presentation and severity
Manuscript number (if known): 151777-INS-CMED-TR-2

In the interest of transparency, we ask you to disclose all relationships/activities/interests listed below that are related to the content of your manuscript. "Related" means any relation with for-profit or not-for-profit third parties whose interests may be affected by the content of the manuscript. Disclosure represents a commitment to transparency and does not necessarily indicate a bias. If you are in doubt about whether to list a relationship/activity/interest, it is preferable that you do so.

The following questions apply to the author's relationships/activities/interests as they relate to the current manuscript only.

The author's relationships/activities/interests should be defined broadly. For example, if your manuscript pertains to the epidemiology of hypertension, you should declare all relationships with manufacturers of antihypertensive medication, even if that medication is not mentioned in the manuscript.

In item #1 below, report all support for the work reported in this manuscript without time limit. For all other items, the time frame for disclosure is the past 36 months.

		**Name all entities with whom you have this relationship or indicate none (add rows as needed)**	**Specifications/Comments (e.g., if payments were made to you or to your institution)**
		**Time frame: Since the initial planning of the work**	
1	All support for the present manuscript (e.g., funding, provision of study materials, medical writing, article processing charges, etc.) **No time limit for this item.**	the Antwerp University Research COVID-19 fund (BOF COVID-19; n° 42830)	
		the Research Foundation Flanders (FWO) COVID-19 funds (n° G0H3220N).	
		**Time frame: past 36 months**	
2	Grants or contracts from any entity (if not indicated in item #1 above).	see 1	
3	Royalties or licenses	___X_ None	

4	Consulting fees	___X_ None	
5	Payment or honoraria for lectures, presentations, speakers bureaus, manuscript writing or educational events	__X__ None	
6	Payment for expert testimony	___X_ None	
7	Support for attending meetings and/or travel	___X_ None	
8	Patents planned, issued or pending	___X_ None	
9	Participation on a Data Safety Monitoring Board or Advisory Board	__X__ None	
10	Leadership or fiduciary role in other board, society, committee or advocacy group, paid or unpaid	__X__ None	
11	Stock or stock options	___X_ None	
12	Receipt of equipment, materials, drugs, medical writing, gifts or other services	__X__ None	
13	Other financial or non-financial interests	__X__ None	

Please place an "X" next to the following statement to indicate your agreement:

__X_ I certify that I have answered every question and have not altered the wording of any of the questions on this form.

ICMJE DISCLOSURE FORM

Date: 14 August 2021
Your Name: Benedicte Y. De Winter
Manuscript Title: A dynamic mucin mRNA signature associates with COVID-19 disease presentation and severity
Manuscript number (if known): 151777-INS-CMED-TR-2

In the interest of transparency, we ask you to disclose all relationships/activities/interests listed below that are related to the content of your manuscript. "Related" means any relation with for-profit or not-for-profit third parties whose interests may be affected by the content of the manuscript. Disclosure represents a commitment to transparency and does not necessarily indicate a bias. If you are in doubt about whether to list a relationship/activity/interest, it is preferable that you do so.

The following questions apply to the author's relationships/activities/interests as they relate to the current manuscript only.

The author's relationships/activities/interests should be defined broadly. For example, if your manuscript pertains to the epidemiology of hypertension, you should declare all relationships with manufacturers of antihypertensive medication, even if that medication is not mentioned in the manuscript.

In item #1 below, report all support for the work reported in this manuscript without time limit. For all other items, the time frame for disclosure is the past 36 months.

		Name all entities with whom you have this relationship or indicate none (add rows as needed)	Specifications/Comments (e.g., if payments were made to you or to your institution)
**Time frame: Since the initial planning of the work**			
1	All support for the present manuscript (e.g., funding, provision of study materials, medical writing, article processing charges, etc.) **No time limit for this item.**	the Antwerp University Research COVID-19 fund (BOF COVID-19; n° 42830)	
		the Research Foundation Flanders (FWO) COVID-19 funds (n° G0H3220N).	
**Time frame: past 36 months**			
2	Grants or contracts from any entity (if not indicated in item #1 above).	see 1	
3	Royalties or licenses	patent (WO/2021/013479)	

4	Consulting fees	___X_ None	
5	Payment or honoraria for lectures, presentations, speakers bureaus, manuscript writing or educational events	__X__ None	
6	Payment for expert testimony	___X_ None	
7	Support for attending meetings and/or travel	___X_ None	
8	Patents planned, issued or pending	patent (WO/2021/013479)	
9	Participation on a Data Safety Monitoring Board or Advisory Board	__X__ None	
10	Leadership or fiduciary role in other board, society, committee or advocacy group, paid or unpaid	__X__ None	
11	Stock or stock options	___X_ None	
12	Receipt of equipment, materials, drugs, medical writing, gifts or other services	__X__ None	
13	Other financial or non-financial interests	__X__ None	

Please place an "X" next to the following statement to indicate your agreement:

__X_ I certify that I have answered every question and have not altered the wording of any of the questions on this form.
